# More than urns: A multi-method pipeline for analyzing cremation burials

**DOI:** 10.1371/journal.pone.0289140

**Published:** 2023-08-30

**Authors:** Lukas Waltenberger, Marjolein D. Bosch, Michaela Fritzl, André Gahleitner, Christoph Kurzmann, Maximilian Piniel, Roderick B. Salisbury, Ladislav Strnad, Hannah Skerjanz, Domnika Verdianu, Christophe Snoeck, Fabian Kanz, Katharina Rebay-Salisbury

**Affiliations:** 1 Austrian Archaeological Institute, Austrian Academy of Sciences, Vienna, Austria; 2 Unit of Forensic Anthropology, Center for Forensic Medicine, Medical University of Vienna, Vienna, Austria; 3 Clinical Division of Radiology, University Clinic of Dentistry, Medical University of Vienna, Vienna, Austria; 4 Clinical Division of Conservative Dentistry, University Clinic of Dentistry, Medical University of Vienna, Vienna, Austria; 5 Center of Clinical Research, University Clinic of Dentistry, Medical University of Vienna, Vienna, Austria; 6 Department of Archaeology, Faculty of Arts, Comenius University, Bratislava, Slovakia; 7 Laboratories of the Geological Institutes, Faculty of Science, Charles University, Prague, Czech Republic; 8 Multidisciplinary Archaeological Research Institute, Department of Art Sciences and Archaeology, Vrije Universiteit Brussel, Brussel, Belgium; 9 Research Unit: Analytical, Environmental & Geo-Chemistry, Department of Chemistry, Vrije Universiteit, Brussel, Brussel, Belgium; University of Padova: Universita degli Studi di Padova, ITALY

## Abstract

Burial rites of archaeological populations are frequently interpreted based on cremated remains of the human body and the urn they were deposited in. In comparison to inhumations, information about the deceased is much more limited and dependent on fragmentation, selection of body regions, taphonomic processes, and excavation techniques. So far, little attention has been paid to the context in which urns are buried. In this study, we combined archaeological techniques with anthropology, computed tomography, archaeobotany, zooarchaeology, geochemistry and isotopic approaches and conducted a detailed analysis on a case study of two Late Bronze Age urns from St. Pölten, Austria (c. 1430 and 1260 cal. BCE). The urns were recovered *en-bloc* and CT-scanned before the micro-excavation. Osteological and strontium isotope analysis revealed that the cremated remains comprised a young adult female and a child that died at the age of 10–12 years. Both individuals had been subject to physiological stress and were likely local. Animal bones burnt at different temperatures suggested different depositional pathways into the urn and pit as part of the pyre, food offerings, and unintentional settlement debris. Eight wild plant and five crop plant species appeared as part of the local landscape, as food offerings and fire accelerants. Sediment chemistry suggests that pyre remains were deposited around the urns during burial. Multi-element geochemistry, archaeobotany, and zooarchaeology provide insights into the Late Bronze Age environment, the process of cremation, the gathering of bones and final funerary deposition.

## Introduction

Archaeological museum collections in Europe house large numbers of vessels that were used as containers for cremated human remains during the Late Bronze Age. Urns from antiquarian excavations, on which such collections were built, were treated as collectible items interesting for their artistic and cultural-historical value [1]. Little attention was paid to the remains of the people within the urns and to the contextual information in the soil surrounding the urn. This has recently changed, with research focusing on the analysis of cremated human remains [e.g. 2–8]. This study expands on this trend in funerary archaeology of cremations with a multi-method approach.

Cremated human remains provide unique analytical and interpretative challenges. Exposure to fire, the subsequent selection of skeletal elements for burial, taphonomic processes, and excavation techniques cause bones to fracture into smaller elements [9, 10] and reduce the amount of morphological information obtained about the deceased. Moreover, ancient proteins and DNA disintegrate above a certain temperature [11]. Further, cremated human individuals are often found commingled with the remains of others, especially when a communal pyre place is preferred, as well as with burnt animal bone fragments resulting from meat offerings cremated together with the dead [12–15].

This article presents the results of a multi- and interdisciplinary approach to the analysis of urn burials. Large cemeteries containing several hundreds of urns of the Late Bronze and Early Iron Ages (c. 1300–600 BCE) gave rise to the name ‘Urnfield Culture’ for the Late Bronze Age in Central Europe. The dead were cremated and usually buried in individual urns, although multiple individuals cremated and buried together are not uncommon [8, 16, 17]. Combining archaeology with osteology, computed tomography, archaeobotany, zooarchaeology, geochemistry and isotopic approaches allows insights into the lives of the buried persons as well as a detailed reconstruction of funerary rituals of the Late Bronze Age, the latter of which is only possible with large samples. As an example, we applied these methods to a case study of two Late Bronze Age urns to present the power of this multimethod approach.

## Materials and micro-excavation

**Fig 1:**
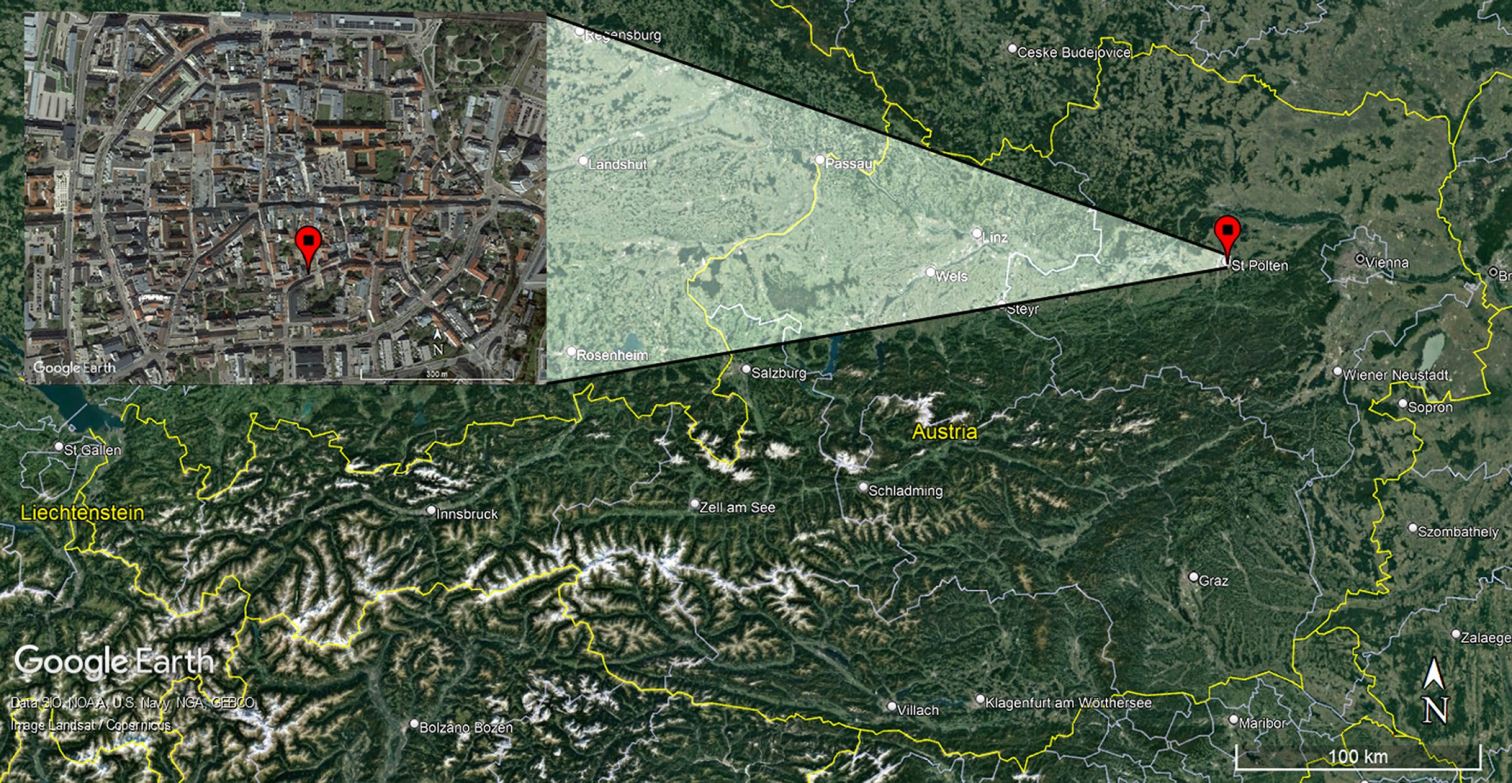
Map of Austria with the marked position of the city St. Pölten and the location of the excavation site Furhmanngasse 3-7in St. Pölten.

The two Bronze Age urns in this study were found in the course of rescue excavations in the historical city center of St. Pölten, Fuhrmannsgasse 3–7, in 2021, amongst other prehistoric, Roman, medieval and modern findings. St. Pölten is situated in the valley of the Traisen river, a tributary to the Danube approximately 50 km west of Vienna in Lower Austria (Fig 1). Large cemeteries such as Early Bronze Age Franzhausen and Gemeinlebarn [18, 19], as well as Late Bronze Age Franzhausen-Kokoron and Inzersdorf [20, 21], are also part of this rich archaeological landscape [22, 23].

**Fig 2:**
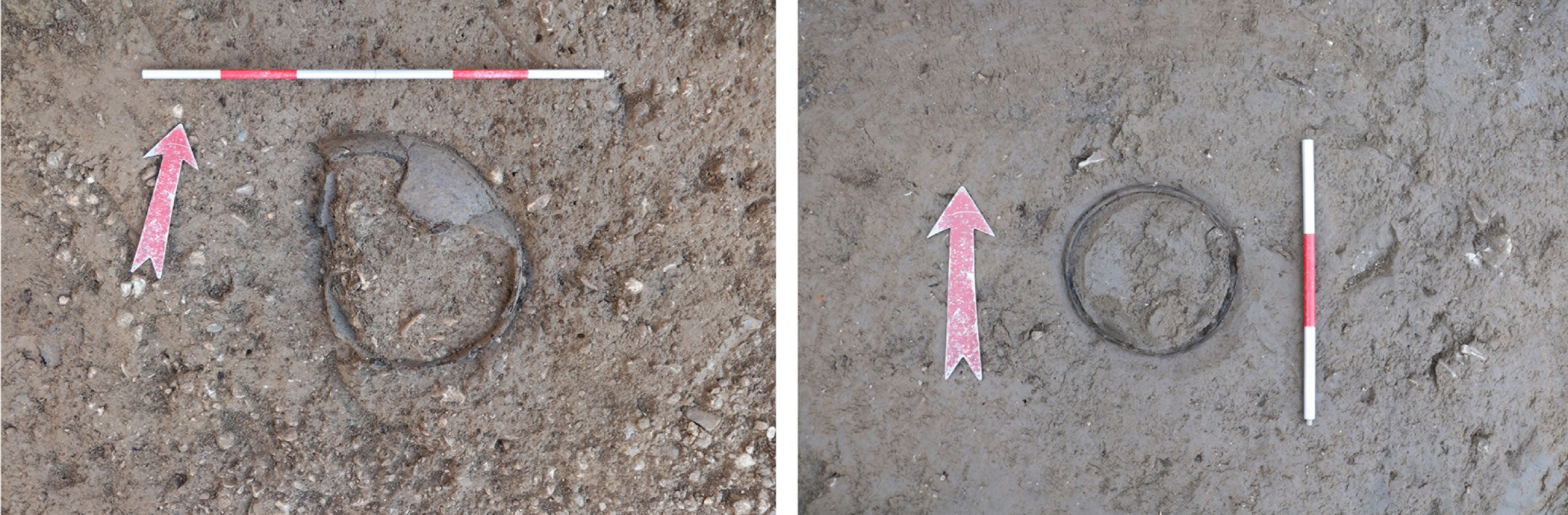
Urn 1 (left) and Urn 2 (right) before recovery. Scale length is 60 cm on the left photo and 30 cm on the right photo.

Urn 1 (SE3605) was found disturbed and damaged at the top, whereas the rim of Urn 2 (SE3602) was complete and discovered about 8 m towards the north-west of Urn 1. The grave pits were not visible in the light brown, silty clay soil during the excavation (Fig 2). Both urns were surrounded with plaster on site and recovered *en-bloc*. A third urn found in the vicinity (SE3632) was poorly preserved and is not included in this study.

**Fig 3:**
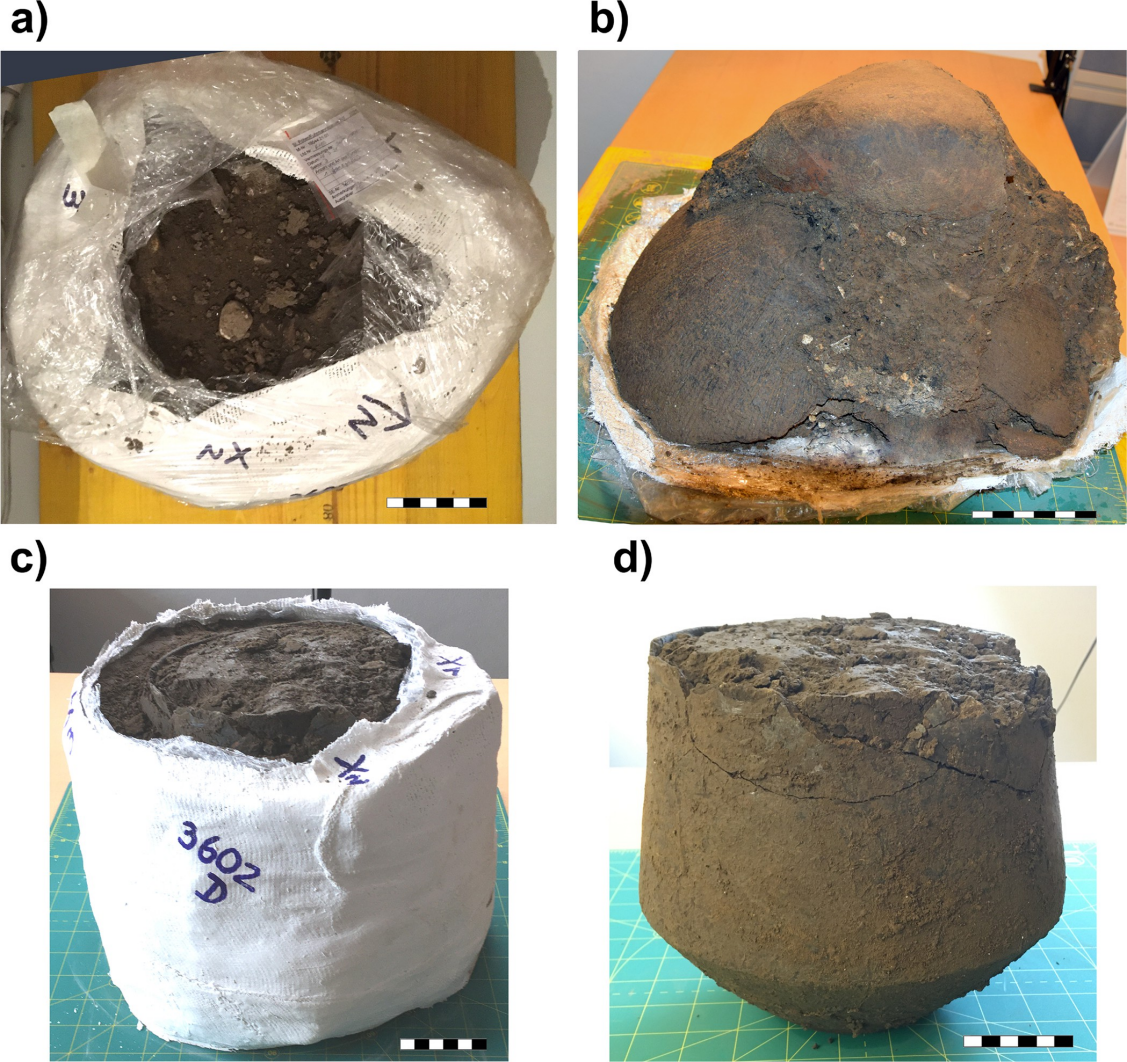
a) Urn 1 after *en-bloc* recovery. b) Urn 1 after removing the plaster and placing it upside down. Please note a part of a second vessel at the top of the block. c) Urn 2 after *en-bloc* recovery. d) Urn 2 after removing the plaster. Scale length is 1 cm.

Still in plaster, the urns were CT-scanned at the University Clinic of Dentistry of the Medical University of Vienna. After digital reconstruction of the content of the urns, they were micro-excavated at the Austrian Archaeological Institute (Fig 3). We used two different excavation techniques for each urn for comparison: Urn 1 was turned upside down; the ceramic sherds were removed, and the urn content was micro-excavated from the base to the upper surface. This reverse technique was chosen to avoid uncontrolled damage to the block and for ease of access. Urn 2 was excavated from the top to the bottom in 1 cm thick arbitrary layers. After the removal of the plaster, the first calcined bone fragments were already visible at the top of Urn 1. Urn 1 was excavated in 20 mm arbitrary layers. All layers were further divided into four quadrants based on cardinal points (NE, SE, SW, NW) to further analyze the vertical and horizontal distribution of the recovered bones. Layers were regularly compared to the CT scans to evaluate discrepancies between the digital and physical excavation. Diagnostic elements that were essential for the anthropological analysis of the cremated remains were documented *in situ* before recovery. Bone fragments in Urn 1 were additionally consolidated *in situ* with an 8% Paraloid^TM^ B72 acetone solution to avoid breakage during recovery. Paraloid B72 is commonly used as a consolidant for bones or ceramics in archaeological conservation [24–26]. Paraloid may affect chemical analysis [27, 28]. Consequently, untreated bone fragments have been chosen for isotope analysis. Furthermore, all bone fragments that were treated with Paraloid were packed separately from the other bones to highlight their consolidation to future researchers. As a comparison, bone fragments from Urn 2 were left untreated.

Soil samples were taken from inside and outside both urns for geochemical analysis. 23.5 liters of soil were used for archaeobotanical analysis. All recovered bone fragments were washed in water and dried for the osteological analysis. One completely calcined diaphyseal fragment of each urn, which had not been treated with Paraloid was taken for radiocarbon dating and strontium isotope analysis. For each present individual, two dental roots were submitted to tooth cementum annulation analysis. Animal bones were separated from human bones during the osteological analysis based on the morphology of the bone fragments (size, thickness and cross-section of shaft diameter [29, 30]), and subsequent zooarchaeological analysis was carried out using a comparative zooarchaeological reference collection to determine taxa.

## Methods and results

### CT scans

CT scans of urns containing cremated remains have been common in the last decades (e.g. [31, 32]). However, based on the site, the soil quality and the CT scanner used, the quality of the scans was highly variable suggesting that CT scanning is a beneficial and time-saving method, but their potential for anthropological analysis is limited [32, 33]. Consequently, Harvig et al. 2012 [10] suggested using CT scanning as a method to support micro-excavation and that it is best suited to gain an overview of the urn content.

In our study, urns were CT scanned using a Siemens SOMATOM Definition AS CT scanner at the University Clinic of Dentistry of the Medical University of Vienna. The best scanning quality was obtained using a scanning protocol with 140 kV, 420 eff. mAs, a voxel size of 0.6 mm, and a slice thickness of 0.5mm. The CT scanning data were semi-automatically segmented after the protocols of Spoor, Zonneveld [34] in the software Amira v6.7 [35]. For this, average Houndsfield units were calculated for different materials and bone fragments of different thicknesses to handle the partial volume effect. Based on these thresholds, the materials were automatically segmented in Amira. Because of irregularities in the scan quality due to artifacts and noise, the segmentation was manually corrected in areas where the automatic segmentation failed. 3D models of the urn, artifacts, identified bone fragments, and general bone fragments were exported in an STL format (.stl) to obtain a first impression of the condition of the urn content. The urn was divided into a series of axial 1 cm thick layers based on the planned excavation layers and the images were post-processed using the Maximum Intensity Projection (MIP) method. Photogrammetric models (.obj) were obtained of the uncovered urns and during several excavation steps using a Nikon DSLR-camera D5300 equipped with an AF-S Nikkor DX 18–105 mm/3.5–5.6G ED VR lens and the software Autodesk ReCap Photo v21.1.3.41.

The CT scans of Urn 1 showed a lower image quality than Urn 2, which was compromised by noise and some cone beam hardening artifacts in the scans. The recovered block was much larger (Urn 1: approx. 460 × 380 mm, Urn 2: 300 × 310 mm) and the soil was very rich in pebbles, which absorbed X-rays during the CT scanning. Moreover, both urns contained metal objects, grave or pyre goods, that were clearly visible on the CT scans.

The density of Urn 1 appeared similar to the surrounding soil in the CT scans, which required manual segmentation of the urn. In areas where the soil was very compact, the shape of the urn vessel was only recognizable as a shadow. The base of Urn 1, for instance, was completely invisible. In other areas, a small air gap developed between the urn and the soil as water evaporated after recovery, which made it easier to distinguish the urn from the surrounding soil. One large ceramic sherd (c. 180 × 140 mm) was visible under the base of Urn 1, which did not belong to the urn based on shape. Urn 1 contained an inhomogeneous layer of cremated bones that sloped up at the western side, whereas the eastern side only contained a thin layer of bones and some bone fragments in the soil above this layer. Since the urn had been placed horizontally in the burial pit, this suggests that after the cremation, the bones were gathered predominantly into one side of the urn; alternatively, the urn could have been held and carried in a tilted way shortly before it was buried. Two large diaphyseal fragments with concentric heat fractures, a distal femur fragment of 120 mm and a humerus fragment of 68 mm, as well as a proximal femoral end were clearly identifiable in the CT scans.

**Fig 4:**
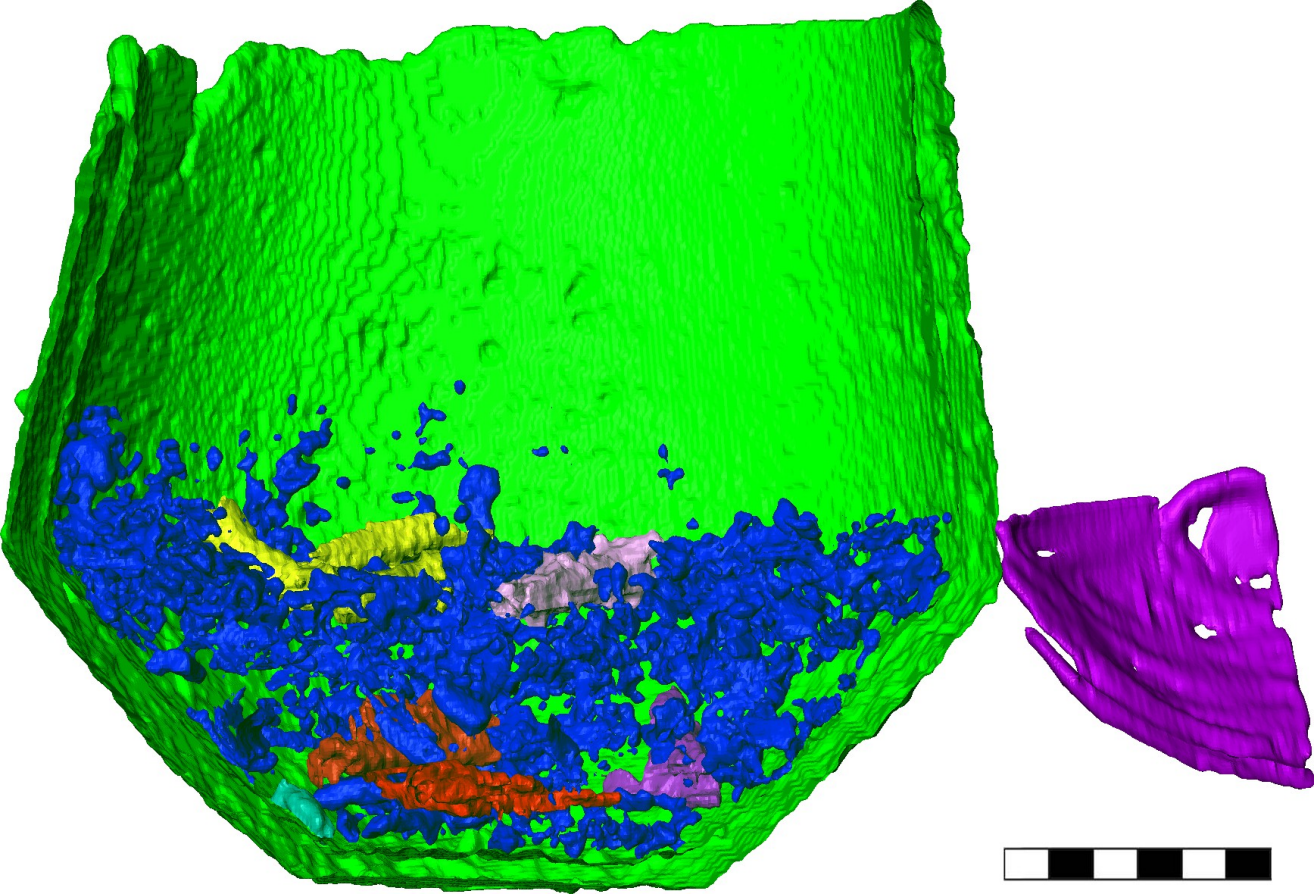
3D surface construction of the findings of Urn 2. Green: ceramic of Urn 2, purple: ceramic sherds of another vessel next to Urn 2; blue: general bone fragments; other colors: identifiable bone fragments. Scale length is 6 cm.

**Fig 5:**
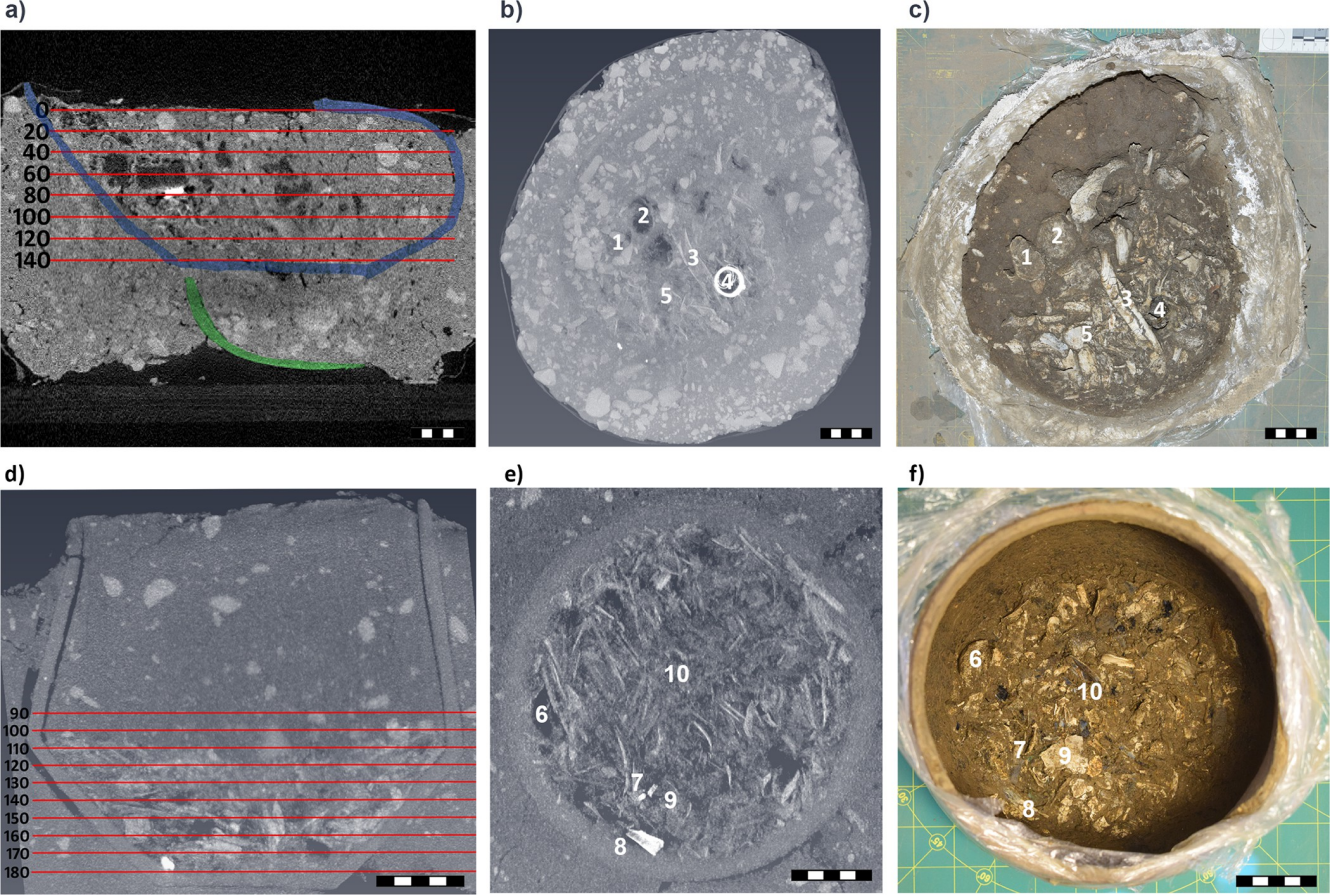
a) mid-sagittal section of Urn 1 with arbitrary excavation layers, b) MIP-projection of layer 80, c) photo of layer 80 during excavation, d) mid-sagittal section of Urn 2 with arbitrary layers, e) MIP-projection of layer 150, f) photo of layer 150 during excavation. The numbers represent identifiable structures in b) and c) or e) and f): 1) lumbar vertebra, 2) femoral head, 3) humerus diaphysis, 4) bronze wire and pendant, 5) cranial fragment, 6) humerus head, 7) bronze spirals, 8) bronze sheet, 9) cranial fragment, 10) tibial fragment. Scale length is 5 cm.

In the CT scans of Urn 2, the urn was easy to distinguish from the surrounding soil, which made the segmentation process straight forward (Fig 4). The scans revealed a layer of cremated bone fragments within Urn 2 that filled approximately one-third of the urn. In comparison to Urn 1, the bone fragments appeared more gracile and had smaller epiphyses. Tibia, femur, humerus, and cranial fragments were identifiable based on the shape of the bone fragments (Fig 5). In addition, one isolated humerus head was present, but the open epiphysis was not explicitly recognized due to insufficient scanning resolution.

### Micro-stratigraphy

**Fig 6:**
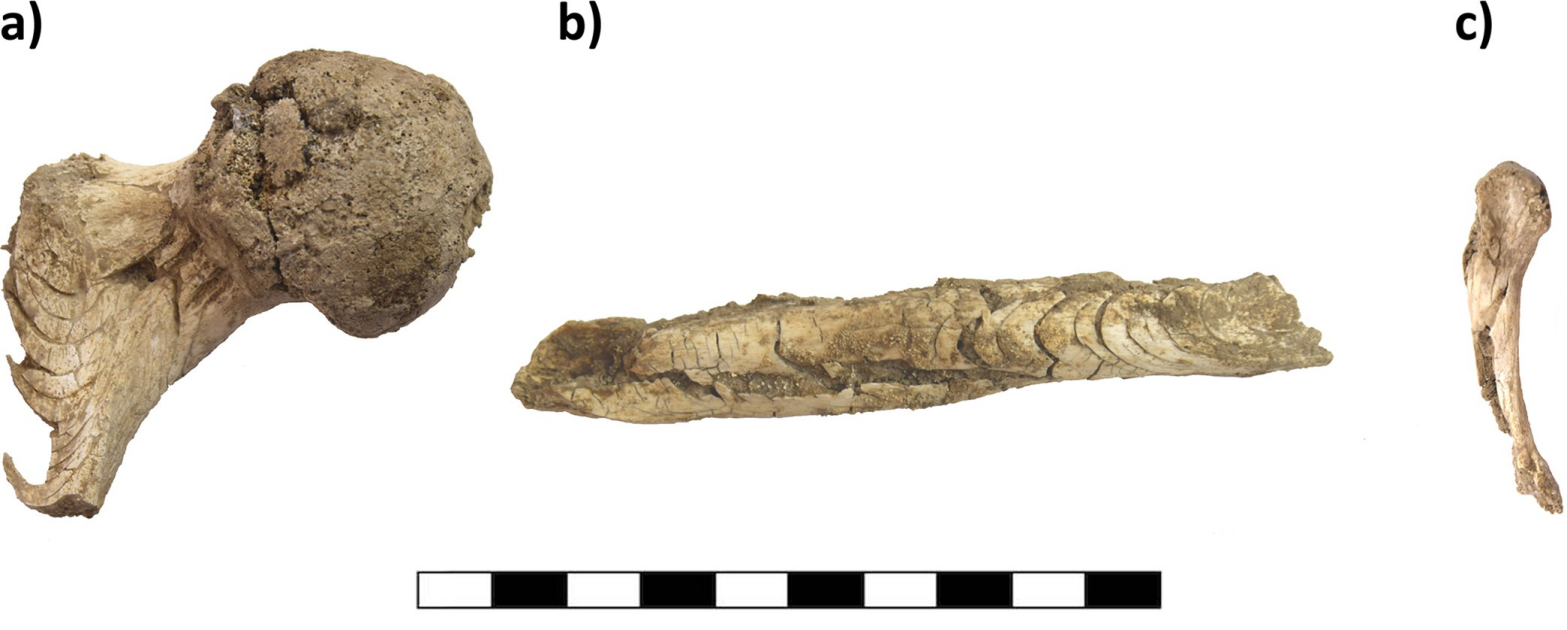
Consolidated bones from Urn 1. a) prox. femoral end, b) humerus diaphysis, c) 4^*th*^ metacarpal bone. Scale length is 10 cm.

During the micro-excavation, we recovered several white to dark grey layers containing ash, some diaphyseal and rib fragments measuring up to 30 mm, calcined bone fragments smaller than 1 mm, small bronze spiral fragments and charcoal under the base of Urn 1. These layers were always observed close to large, old cracks in the urn, indicating that water had washed these fragments out over time. After removing the sherds of the urn, the Urn 1 content was micro-excavated in seven 2 cm arbitrary layers. Several well-preserved elements were identified that were not visible in the CT scans, for example a mandibular body with premolar and molar roots still in the dental sockets, an acetabulum, several complete vertebral bodies, several metacarpal bones, and both proximal femoral ends, that are rarely preserved intact in prehistoric cremated remains. All elements which would fracture into non-recognizable fragments after recovery were stabilized using Paraloid (Fig 6). Before the application of Paraloid, the bone surface was carefully cleaned with water and cotton swabs to allow a better impregnation of the bones with the consolidant. After the water evaporated, the 8% solution was applied with a pipette. For 5 minutes, the bone was dried until the surface was not sticky anymore. The bone was recovered and extra Paraloid and soil particles sticking to the bone surface were carefully wiped off using cotton swabs and acetone.

**Fig 7:**
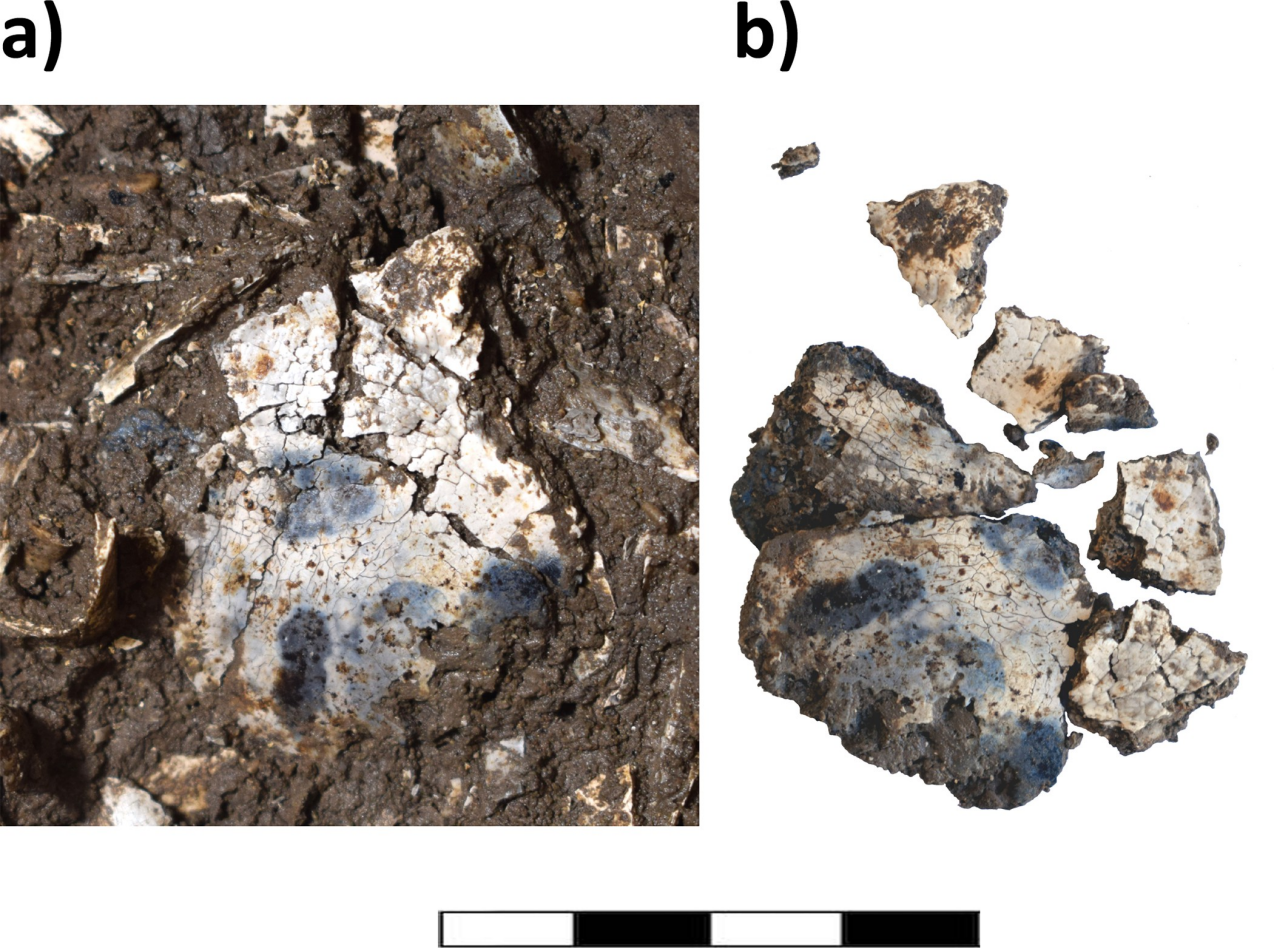
Cranial fragment from Urn 2. a) *in situ* fragmented cranial bone and still in the original shape, b) after removal, the bone fragments fell apart which affects the anthropological analysis. Scale length is 4 cm.

Urn 2 was completely intact except for a partially fragmented rim and a crack that split the urn in half along the sagittal axis. During the removal of the soil surrounding Urn 2, a base fragment of a thin-walled ceramic vessel was recovered towards the north side. The vessel was only visible as a weak shadow in the CT scans and would have been easily overlooked. To avoid breakage during micro-excavation of the urn content, the urn was wrapped in cling film and excavated in nine 1 cm layers. The upper two thirds of the urn were filled with homogenous soil containing only a few pebbles. Diaphyseal fragments, tooth roots and several cranial fragments were present, which could not be easily recognized on the CT scans. We did not stabilize the bones of Urn 2 prior to removal to test differences in the recovery of bone fragments which were consolidated or remained untreated, but documented bone elements *in situ* (Fig 7).

### Bio-anthropological analysis

Following the guidelines by Brickley and McKinley [36] and Jaskulska [37], fragments were washed and sieved into 10 mm, 5 mm, 2 mm and <2 mm fractions and separated into the body regions (cranium, axial skeleton, upper, and lower limbs) based on morphological features. The bone distribution in quadrants and arbitrary layers were analyzed using the Kruskal-Wallis test with Monte-Carlo correction and Dunn-Bonferroni test for post-hoc pairwise testing. Significant *p*-values were adjusted using the Bonferroni correction. For each body region, the largest fragment size was measured. General bone fragments were further divided into diaphyses, epiphyses, autopodia, and rest.

The type of visible heat cracks and the coloration of the bone fragments were recorded separately for each body area. The burning temperature was estimated based on Wahl [38]. Dentition was evaluated based on the FDI system following the coding system of Harbeck [39]. Sex was estimated using morphological methods for cranium and pelvis [40, 41], and metric measurements were taken for the postcranial skeleton [42]. Age at death was estimated based on epiphyseal closure [43], tooth eruption [44], the iliac auricular surface [45, 46], cranial suture closure [47], and tooth cementum analysis (adapted from Wittwer-Backofen et al. 2004, Naji et al. 2016). Enthesopathies were scored based on Mariotti et al. [48].

Urn 1 contained 1149.56 g of calcined human bone fragments and 20.49 g burnt animal remains. The degree of fragmentation was low with nearly 60% of all fragments measuring over 10 mm and only 15.7% of all fragments measuring less than 5 mm in length. The largest fragment is 111 mm long. The osteological examination revealed that bones from all body areas were present in the cremated remains. Nearly three quarters of all bone fragments were identified and assigned to a body region based on morphological features (Table 1).

**Table 1:** Weight of body regions and sieve fractions of the cremated remains recovered from Urn 1.

[g]	∑	≥ 10 mm	5–10 mm	2–5 mm	≤2 mm
**Cremation weight**	1149.56	677.04 (58.9%)	291.4 (25.3%)	93.21 (8.1%)	87.91 (7.6%)
**Cranium**	218.88 (19.0%)	110.53	78.09	30.26	-
**Axial**	301.61 (26.2%)	204.17	75.50	21.94	-
**Upper limbs**	114.46 (10.0%)	106.83	7.10	0.53	-
**Lower limbs**	196.78 (17.1%)	179.13	17.17	0.48	-
**Diaphyses**	162.49 (14.1%)	65.76	74.83	21.90	-
**Epiphyses**	30.29 (2.6%)	6.72	17.36	6.21	-
**Autopodia**	8.28 (0.7%)	1.03	5.67	1.58	-
**Rest**	116.77 (10.2%)	2.87	15.68	10.31	87.91

A detailed analysis of the vertical and horizontal distribution of body areas was not possible. A quantitative comparison of the four quadrants revealed that the bones were not evenly distributed in the urn, with the fewest bone fragments in the northern and eastern quadrants and a gradual increase from north-east (294 g) to south-west (749 g) within the urn (see S1 Fig), which was already visible in the CT scans.

The remains in Urn 1 were predominantly well burnt with the white color of completely calcined bones dominating over bones presenting grey or black areas (burning stage V after Wahl [38]). Additionally, the burning condition was also analyzed using a Fourier Transform Infrared Spectroscopy (FTIR) following the protocols of Stamataki et al. [49]. No organic matter was visible in the infrared spectra and the infrared splitting factors (IRSF) which provides information on the bone apatite crystallinity which measured 4.5 in urn 1, indicating the sample was completely calcified. Typical heat fractures such as warping, delamination, longitudinal, transverse, and parabolic fractures were visible. This is consistent with burning temperatures over 800°C. Green staining was observed on the sacrum and several rib and arm fragments, which is often associated with bronze artifacts close to these bone elements.

Overall, many body areas were well represented in the cremated remains from Urn 1. The neurocranium was well preserved with the most important morphological structures present. Several dentin fragments and tooth roots were recovered, of which only a few premolars and molars could be further identified. There were no tooth crowns present. Several sternal and vertebral rib ends, vertebral bodies from all over the spine, the sacrum and the ilium including the greater sciatic notch and one auricular facet were identifiable from the axial skeleton. The upper limbs were represented by a humerus head, further fragments from humerus, ulna and radius, and several hand bones including carpals, metacarpals, and hand phalanges. Identified elements of the lower limbs included left and right proximal femoral ends, fragments of femur, tibia and fibula, the right talus, one first metatarsal bone, and further foot bones.

The sex of the deceased individual was estimated as female based on morphological features of cranium and coxa and a metric evaluation of the mandibular condyle, radial head, and femoral head (prob_female_ = 0.96). Age at death of 23–32 years was estimated based on the iliac auricular surface and the transitional analysis using both cranial structures and the iliac auricular surface (mean = 29 years, age category: adult), and a tooth cementum analysis (26.6 years; ± 5 years). Cribra cranii is visible at the external surface of the cranial vault. At the ilium, the preauricular sulcus is present. Overall, the skeleton is gracile and muscle attachments are weakly developed, except for the attachment side of the deltoid muscle (R3). A few cremated remains recovered from Urn 1 clearly did not belong to the individual described above, namely two fully erupted premolars of an individual older than 15 (FDI 24 and 34 were present twice), and a second dens axis. The premolars were also cremated and only the roots were present.

Urn 2 totaled 696.37 g of burnt human remains. 40% of all bone fragments measured between 5 and 10 mm and 38% of all bone fragments were unidentifiable. The longest fragment measures 65 mm. Bone fragments from all body regions were recovered (Table 2). The horizontal and vertical distribution of bones within the urn did not reveal any trends (S2 Fig). The cremated remains were predominantly calcined and showed heat fracturing such as warping, delamination, longitudinal, transvers, and parabolic fractures. Bone fragments rarely exhibited grey or charcoaled areas (burning stage V after Wahl [38]). Similar to the FTIR results of Urn 1, the analyzed fragment of Urn 2 was also completely calcined with a IRFS value of 4.6. This again suggests a burning temperature over 800°C. Green staining from bronze pyre goods was observed on arm fragments and one cranial fragment.

**Table 2:** Weight of body regions and sieve fractions of the cremated remains recovered from Urn 2.

[g]	∑	10mm	5mm	2 mm	1 mm
**Cremation weight**	696.37	192.33 (27.6%)	278.61 (40.0%)	100.15 (14.4%)	125.28 (18.0%)
**Cranium**	234.42 (33.7%)	69.41	126.25	38.76	-
**Axial**	57.63 (8.3%)	3.38	33.87	20.38	-
**Upper limbs**	49.82 (7.2%)	25.51	23.53	0.78	-
**Lower limbs**	90.99 (13.1%)	77.32	12.95	0.72	-
**Diaphyses**	115.30 (16.6%)	12.59	72.07	30.64	-
**Epiphyses**	9.03 (1.3%)	1.87	5.58	1.58	-
**Autopodia**	0.85 (0.1%)	0.00	0.81	0.04	-
**Rest**	138.33 (19.9%)	2.25	3.55	7.25	125.28

The cremated remains from Urn 2 were more fragmented than those of Urn 1. Nevertheless, several fragments from the neurocranium, viscerocranium, and the mandible were recognized (frontal bone, temporal bone, occipital bone, maxilla, zygomatic bone). Several roots of incisors and one partially erupted lower premolar were identified amongst other tooth fragments present in the urn. Two deciduous molar root fragments were also present. Fragments from vertebrae, ilium, diaphyses from the upper and lower limbs, a sternal rib end, one first metatarsal bone, and three hand phalanges were recognized from the postcranial skeleton.

An age at death of 8.5–14.5 years (mean = 11 years, age category: infans II) was estimated based on tooth eruption and epiphyseal closure. Tooth cementum analysis suggested an age-at-death of 15.0 years (± 5 years). Due to the young age, a sex estimation was not attempted. Active cribra orbitalia, stage 3 after Steckel et al. [50], was observed at the orbital portion of the frontal bone. Cribra cranii affected the occipital bone, and an area of periosteal new bone formation was identified at a diaphyseal fragment of the humerus. Further details on the age at death estimation and sex determination of both individuals can be found in S1 Appendix. The raw data is available in S1 Dataset.

### Sr-isotopes

Following the recent demonstration that calcined bone provides a reliable substrate for strontium isotope analyses [51, 52], strontium isotope ratios (^87^Sr/^86^Sr) of cremated bone samples were pre-treated following Snoeck et al. [52]. Strontium was extracted from the samples and purified following the protocol described in Snoeck et al. [52] and measured on a Nu Plasma 3 MC-ICP Mass Spectrometer (PD017 from Nu Instruments, Wrexham, UK) at the Vrije Universiteit Brussel (VUB). During this study, repeated measurements of the NBS987 standard yielded ^87^Sr/^86^Sr = 0.710240 ±24 (2SD for 60 analyses), which is consistent with the mean value of 0.710252 ±13 (2SD for analyses) obtained by TIMS (Thermal Ionization Mass Spectrometry) instrumentation [53]. All the sample measurements were normalised using a standard bracketing method with the recommended value of ^87^Sr/^86^Sr = 0.710248 [53]. Procedural blanks were considered negligible (total Sr (V) of max 0.02 versus 9–10 V for analyses, i.e. ≈ 0.2%). For each sample the ^87^Sr/^86^Sr value was reported with a 2SE error (absolute error value of the individual sample analysis–internal error). The strontium isotope ratios of the cremated human bones (Table 3) fall in the local range of the river valley of the Traisen [54]. The thresholds of local Sr ratios were assessed based on plant samples collected in different geological zones. There is nothing to suggest other than local origin, although a life in the same geological substratum elsewhere is possible.

**Table 3:** Strontium isotope ratios and C14 dating of the cremated individuals from Urn 1 and 2.

Lab Code	Grave	Element	87Sr/86Sr	2 SE	C14	2 SE
MF213 (Sr) RICH-31797 (C14)	Urn 1 L3W	Humerus	0.708889	0.000020	3068 BP	±25
MF212 (Sr) RICH-31793 (C14)	Urn 2 L4W	Femur	0.708554	0.000007	3100 BP	±26

### Radiocarbon dating and typochronology

**Fig 8:**
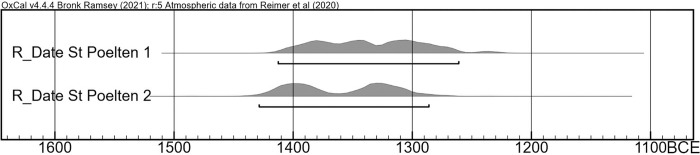
Calibration curve of the radiocarbon dates of cremated bones from Urn 1 and Urn 2.

**Fig 9:**
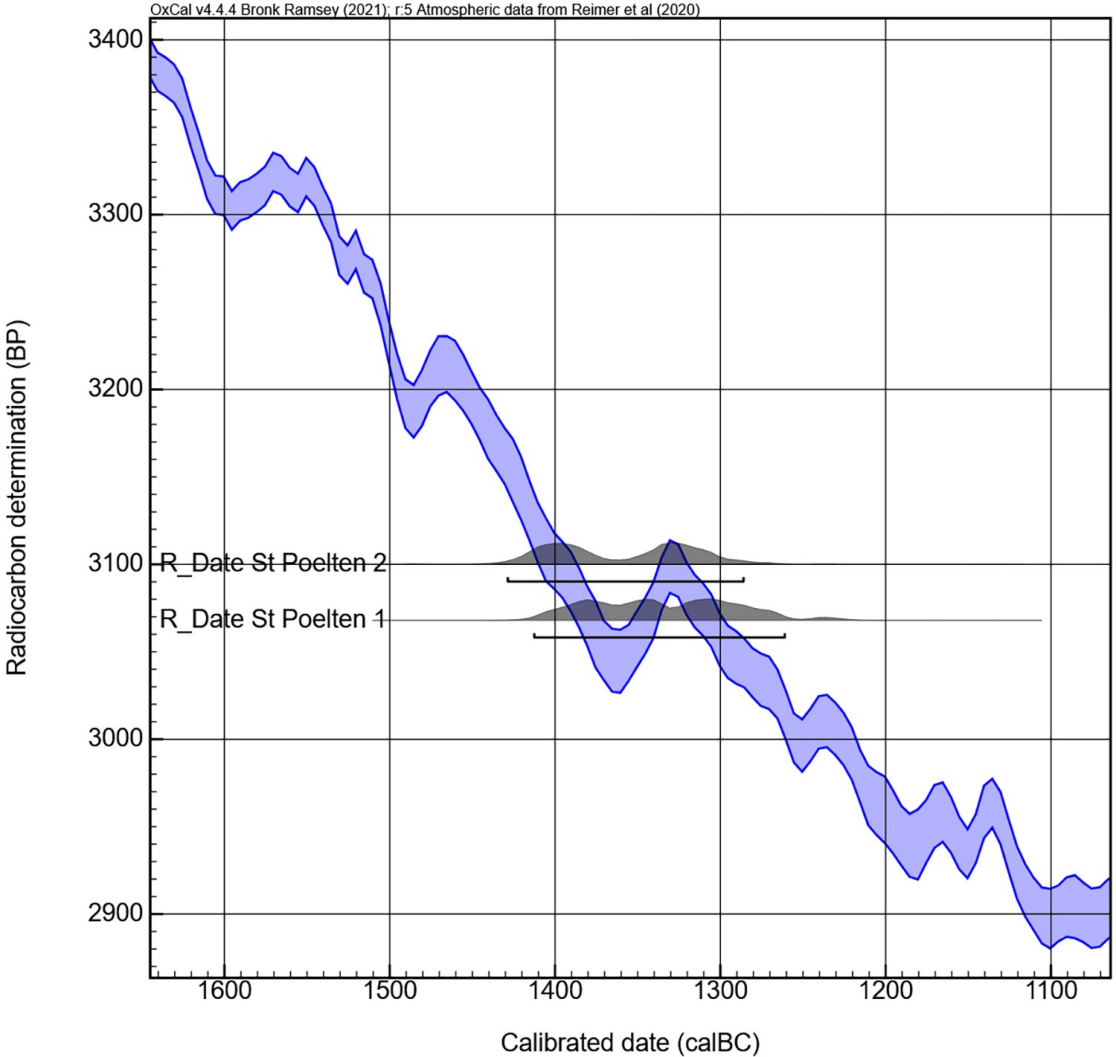
Curve plot from the calibration of the radiocarbon dates of cremated bones from Urn 1 and Urn 2.

The samples of cremated human bone for strontium isotope analysis were split, and the other half was sent to the C14 Laboratory of the Royal Institute for Cultural Heritage in Brussels (Mathieu Boudin) for radiocarbon dating. This should prevent the possibility of the urns containing two individuals which could not be distinguished using anthropological methods as shown by Sabaux et al. [8]. Using one completely calcined bone fragment for C14 and Strontium analysis allows a more detailed interpretation of the results. Urn 1 (RICH-31797) returned a raw date of 3068 ±25 BP, Urn 2 (RICH-31793) of 3100 ±26 BP. With 95.4% probability, this corresponds to the period between 1430 and 1260 cal. BCE (Table 3, Figs 8 and 9).

Urn 2 is considered a classic shape for the early Urnfield Culture (Velatice phase) with its sharp profile, which would conventionally be dated to Bronze Age D, c. 1300–1200 BCE [55]. However, the double conical vessel shape appears early, in the transitional phase between inhumation and cremation at the beginning of the Urnfield Culture [56]. The early radiocarbon dates support the view that the Late Bronze Age began already in the 14^th^ century BCE [57], even taking a possible old wood effect into account as during cremation there is an exchange of carbon between bioapatite and the fuel [58, 59].

### Artifacts

Ceramic and bronze artifacts were recovered with the cremated human remains (see S2 Appendix). The ceramic vessel Urn 1.1 served to contain the cremation. The large storage vessel with comb-stroke decoration was fragmented and deformed, with impact from the western side, and only the bottom part was found. The urn was placed on the fire-affected sherd of another large vessel decorated with wide cannelure (1.2).

A solid cast bronze ring of c. 30 mm diameter (1.3) with oval cross-section and protruding thorn found in the urn was most likely a dress fitting. Comparable rings have been found at Inzersdorf and Franzhausen-Kokoron [14, 60]. A coil *Noppenring* (1.4) made of oval bronze wire was found with 2.5 remaining coils and partly smelted by the fire in the urn. It may have been used as temple, hair or finger ring, and represents a characteristic, long-lived Bronze Age form [e.g. 19, 61]. The fire deformation and the thick, flaky, grey corrosion layer on both artifacts suggest an exposure to temperatures between 700 and 900°C on the pyre [62]. Further bronze wire fragments (1.5–1.7) were found both inside and outside the urn. Tiny molten bronze drops and their remnants (1.8–1.13) were found interspersed with the cremated remains inside and outside the urn.

The bi-conical shape with a distinct profile and sharp bend in the lower part of Urn 2.1 belongs to the early Urnfield Culture phase Bronze Age D [16, 55]. The fragmented remains of a small, fire-affected fine vessel, probably a cup or bowl, were found north of the urn and are most visible in the CT scan (2.2). Further small pottery fragments were recovered as part of the fill within and outside the urn (2.3–2.5).

A fragment of a twisted bronze wire found at the bottom of the urn was probably part of an arm ring (2.6), with a shape typical for southern Germany and Austria from the Middle Bronze Age to the Urnfield Period [63]. The grey, slate-like corrosion layer suggests the object was worn on or near the body during cremation [64]. A conical object made of folded sheet bronze (2.7) with signs of deformation caused by the funerary fire might represent a clasp used to cover the ends of cords or textiles [21, 65]. Fragments of two different types of beads made of bronze wire coils were recovered among the human remains in different locations within the urn (2.8–2.10). A bronze drop (2.11) from an artifact molten on the pyre was found at the western base part of the urn. The catalogue of finds is published as S2 Appendix.

### Zooarchaeological analysis

Urn 2 did not contain any animal remains. In total, 56 animal bone fragments were uncovered from Urn 1, varying in size from 4 to 62 mm (Table 4). The bones showed various taphonomic signatures suggesting several depositional pathways into the urn itself and its context such as meat offerings, bones as fuel, or animal bones as garbage. In addition, a series of natural terrestrial mollusks were recovered from both the sediment inside and outside the urn. About thirty dental fragments including those of an incisor, a canine and a lower premolar of a young adult wild boar (*Sus scrofa*) probably represents a single partial mandible. These fragments show extensive burning damage congruent with the human osteological material. Further, a red deer (*Cervus* elaphus) metapodial fragment and a mammalian rib fragment were completely calcined and like the wild boar mandible were probably part of the pyre. An additional distal scapula fragment of a sheep or goat was only superficially charred on the ventral part of the neck and onset of the blade, probably originating from a meat offering. Several unburnt bones from the grave fill around the urn include a probably naturally shed upper left deciduous third premolar showing root resorption and a fragment of a left humerus of wild boar. These unburned bones show extensive cortical weathering including root etching and pitting and appear to be domestic waste within the sediment used to cover the urn.

**Table 4:** Inventory of the recovered animal bones from Urn 1.

element	species	sex	age at death	layer	burning condition	interpretation
**shells**	terrestrial mollusks	-	-	all layers inside and outside the urn	unburnt	local intrusive fauna
**canine, lower premolar, incisors**	wild boar (*Sus scrofa*)	male	young adult	inside urn, upper west	extensive	part of pyre
**rib fragment**	wild boar-ibex size	-	-	inside urn, center east	extensive	part of pyre
**metapodial fragment**	red deer (*Cervus elaphus*)	-	-	inside urn, upper west	extensive	part of pyre
**distal scapula fragment**	sheep or goat	-	adult	inside urn, upper west	superficially charred	meat offering
**upper left deciduous third premolar**	wild boar (*Sus scrofa*)	-	subadult (extremely worn; root resorption)	exterior	unburnt, cortical weathering	part of the grave fill
**left humerus**	wild boar (*Sus scrofa*)	-	-	exterior	unburnt, cortical weathering	part of the grave fill
**diaphyseal fragment**	wild boar-ibex size	-	-	-	unburnt, cortical weathering	part of the grave fill

### Archaeobotanical analysis

Urn content, the soil around the urns and the ash layer under Urn 1 were analyzed separately. Plant remains and other floating components were extracted through flotation [66] of the material using sieves with a mesh size of 0.5 and 1 mm. After drying, the components were sorted into charred diaspores, chaff, charcoal, and recent plant residue under a stereomicroscope. Present taxa were identified and counted.

**Fig 10:**
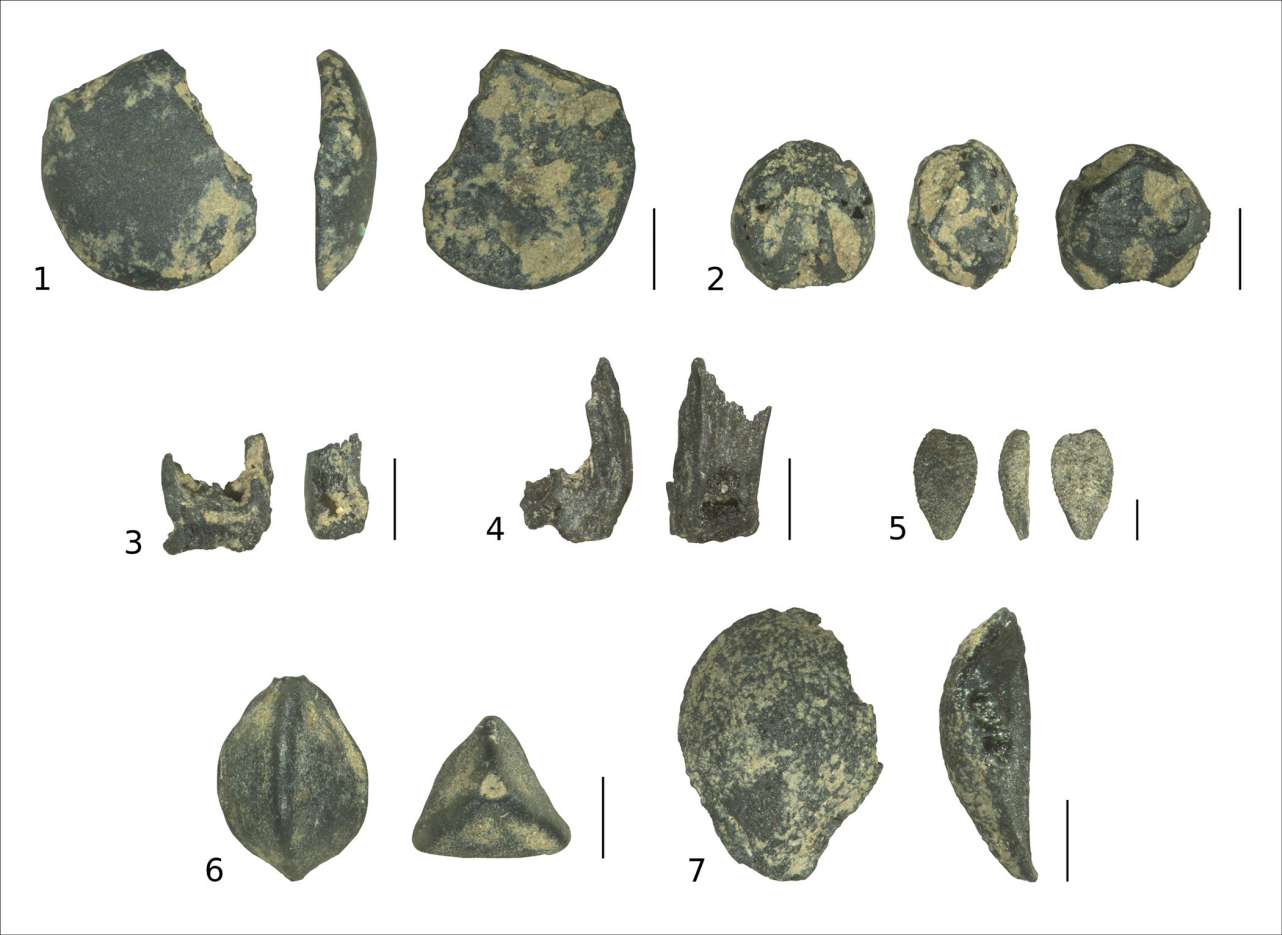
Charred seeds and chaff from Urn 1: crops: 1 lentil (*Lens culinaris*), 2 common millet (*Panicum miliaceum*); chaff: 3 einkorn (*Triticum monococcum*), 4 emmer (*Triticum dicoccum*); wild plants: 5 black elderberry (*Sambucus nigra*), 6 black-bindweed (*Fallopia convolvulus*), 7 white bryony (*Bryonia alba*). Scale length is 1 mm.

A total of 19 036 carbonized plant remains were recovered from Urn 1 (Table 5). All except 189 could be attributed to the goosefoot family (18844 *Chenopodium sp*., 3 *Chenopodium hybridum*). The rest could be assigned to 11 taxa (6 wild plants, 5 crops; Fig 10). The large number of identified chaff from einkorn (*Triticum cf*. *monococcum*) and emmer (*Triticum dicoccum*) is remarkable (*n* = 123). As a crop plant, only one lentil (*Lens culinaris*) was identified from within Urn 1. Seeds from three further crop plants were recovered from the surrounding soil: emmer, spelt (*T*. *spelta*), and common millet (*Panicum miliaceum*). Recovered wild plants were black-bindweed (*Fallopia convolvulus*), false cleavers (*Galium spurium*), yellow bedstraw (*Galium verum*), small mallow (*Malva pusilla*), white bryony (*Bryonia alba*), and black elderberry (*Sambucus nigra*). From Urn 2, only 200 mL of soil was analyzed, as the remaining soil was rinsed in sieves with a mesh size of 1 mm to recover bone fragments. Only charcoal larger than 1mm was recovered from the soil in urn 2.

**Table 5:** Amount of analyzed soil volume per layer and number of identified plant remains from Urn 1.

	Sum	inside urn	ash layer	grave fill
**Soil volume (l)**	23.45	4.5	0.45	18.5
**Number of plant remains (without charcoal)**	19 036	1 380	198	17 458
**Crop plant remains**	149	27	22	100
**Wild plant remains**	43	3	7	33
Find density (per liter; without C*henopodium sp*.)	8.2	64.7	64.4	7.2

### Geochemical analysis

Best practice recommendations for situations where undamaged urns are recovered include systematic sampling of the grave-pit fill and the fill of the urn for multi-proxy analysis to test the burials for signatures of degraded body tissue and environmental conditions [67]. Samples were ground to analytical fineness, and sub-samples (each 0.5 g) were digested (leached) in 10 mL of mixture of inorganic acids (HNO_3_/HCl 10:1, HNO_3_ 65% (~14M), HCl 37% (12M)) in platinum crucible for 1 hour on a hot plate (90°C) and then transferred to a 50 mL volumetric flask. All the acids used were reagent grade (Merck, Germany and Penta Chrudim, CZ) and double distilled. Purified water obtained from a Millipore system (Millipore, USA) was used for all the dilutions. This digestion was selected for leaching only non-silicate forms of elements from the sediments. The major and trace elements were determined by conventional solution nebulization ICP-OES Agilent 5110 (Agilent Technologies, USA). Four-point calibration curves were done and multi-element stock standard reference solution (Analytika Ltd, CZ) was used. Quality control was assessed using multi-element stock solution Merck (Merck, Germany). Data were processed on-line using Agilent ICP Expert software and corrected for procedural blank. Relative standard deviation (RSD) for individual measurements varied from 0.1 to 3% for most elements.

In Urn 1, median weight % values and standard deviations (SDs) for most elements were higher in the sediments surrounding the urns than those above and with the remains from inside the urns (Table 6). This is true in particular for the elements Al, Ca, Cu, Fe, K, and Mg. P is the exception, with internal samples having lower median values, but a slightly higher mean value and SD compared to external samples. Mean and median values for Ca were slightly higher in the internal samples, but the range is much lower. Sample urn1_11, from the west side of the external upper layer, had unusually high values of Al, Ba, Cu, P, Sr and Zn, and low value for Mg. Other elements in sample urn1_11 fell within Quartile 3 or the upper end of Quartile 2. One other sample from the external upper layer, urn1_08, also had unusually high value for Cu; all other elements were unremarkable. Sample urn1_06 was noted during sampling as having darker color and a greasy texture, but this was not reflected in the ICP-OES results.

**Table 6:** Geochemical sample locations around urn with mean weight % and standard deviations for all analyzed elements.

		Al	Ba	Ca	Cr	Cu	Fe	K	Mg	Mn	Ni	P	Pb	Sr	Zn
**Urn 1 internal**	mean	0.92	0.006	9.107	0.001	0.012	1.501	0.199	4.698	0.059	0.002	0.186	0.001	0.007	0.006
	SD	0.0400	0.0005	0.1495	0.0000	0.0099	0.0242	0.0099	0.1470	0.0058	0.0001	0.1791	0.0001	0.0007	0.0003
**external**	mean	0.97	0.007	9.060	0.002	0.041	1.507	0.192	4.570	0.061	0.002	0.174	0.001	0.008	0.006
	SD	0.1939	0.0037	0.4897	0.0004	0.0748	0.0791	0.0199	0.4446	0.0037	0.0003	0.1625	0.0002	0.0035	0.0011
**Urn 2 internal**	mean	0.806	0.005	10.630	0.001	0.002	1.363	0.169	4.999	0.046	0.002	0.101	0.001	0.009	0.004
	SD	0.0794	0.0001	0.1131	0.0001	0.0001	0.0503	0.0143	0.0313	0.0009	0.0000	0.0069	0.0001	0.0001	0.0001
**external**	mean	0.846	0.005	10.452	0.001	0.005	1.422	0.179	4.806	0.042	0.002	0.085	0.001	0.009	0.004
	SD	0.0868	0.0004	0.3953	0.0001	0.0082	0.0935	0.0152	0.2662	0.0029	0.0001	0.0095	0.0001	0.0005	0.0002

In Urn 2, median weight % and standard deviations for most elements were again higher in the sediments surrounding the urns than those within the urns. Ca was the main exception here, with a mean, median and standard deviation values higher than external samples. Mg, Mn and P gave slightly higher mean and median values from inside the urns, but with lower deviations and a lower range. One sample collected from below the urn (external), urn2_12, had values for Al, Ca, Cu, K, P and Sr that are the maximum values for the Urn 2 samples. For both urns, mean weight % for Cr, Ni, Pb, Sr and Zn were 0.01 or below.

## Discussion

### Comparing CT and micro-excavation data

Previous studies using CT scans of whole urns [e.g. 10, 32, 68, 69] concluded that CT scanning is a time-saving method that allows detailed impressions on aspects of the cremations, such as the recognition of skeletal elements, differentiating adults and subadults, and a preliminary sex estimation. However, thick layers of soil, stones in the fill and metal artifacts reduce the quality and utility of the CT scans. On the one hand, clinical CT scanning adds valuable information to the analysis and allows a better planning of the excavation [cf. cf. 32, 33], but it cannot replace a carefully performed micro-excavation, at least in our case. On the other hand, micro-excavation strongly affects the degree of fragmentation as identifiable bone fragments may break into smaller pieces during recovery causing information loss and bone preservation bias. Therefore, the combination of both methods is recommended. Micro-CT scanning would provide higher resolutions of the urn content and therefore may allow a complete virtual excavation of the urn content. S1 Text provides further details into time investment of the single tasks.

Higgins et al. [31] compared CT scans of a Roman urn burial with micro-excavation came to similar results (time-intensive manual segmentation lead to best results, destructive nature of micro-excavation). However, their urns measured only half the size of our urns, which strongly increased the scan quality. We think if urns will be recovered en-bloc with surrounding soil, a micro-excavation of the attached filling to the urn needs to be conducted prior to CT scans, although it may not be useful in every case, as in strongly fragmented urns, the block may break. Furthermore, Higgins et al.’s study [31] also showed a destruction of diagnostic elements during micro-excavation. They may have been preserved if Paraloid had been used. The similar density of soil and ceramic makes it difficult to evaluate fragmentation and compression state of the urn, at least in our case. Furthermore, gracile bones were often blurred on the CT scans, which made a manual segmentation necessary to obtain 3D meshes. Segmentation is not an asset to evaluate the urn content, as most information can be obtained based on the slices and Maximum Intensity Projection provides 3D information on the bones. Nevertheless, measuring diagnostic elements is much easier and quicker on 3D-surface meshes than on single slices.

Heat alteration of bones such as longitudinal and parabolic heat fractures or warping can be easily assessed in the scanning images. One disadvantage of CT scanning is that the density of the objects is represented by greyscale values and therefore, no true color information about the cremated remains is available, although some studies suggested an evaluation of the burning condition is possible as heat affects the density of the bones [10]. Burning temperature, dependent on many variables such as oxygen availability and exposure time [70], affects bone coloration. More advanced methods like X-Ray Diffraction (XRD; [71, 72]) and Fourier Transform Infrared Spectroscopy (FTIR; [73]) would provide a better impression on the burning conditions.

### Lifeways of two Bronze Age individuals

The small group of Late Bronze Age urn burials included the remains of a 23-35-year-old female individual, a woman of childbearing age, and a 10-12-year-old child, who died in the 14^th^ century BCE. They had most likely lived close to the place they were buried. The zooarchaeological suggests a use of both domestic and wild animals. The spectrum of cultivated plant remains from Urn 1 include lentil, einkorn, emmer, spelt and millet, which fits our knowledge of the Late Bronze Age agriculture in Eastern Austria [74]. Seeds attributed to the goosefoot family, were found in large numbers, probably because a single plant can produce up to 1.5 million seeds [75]. These plants commonly grow at fields and ruderal habitats which may suggest evidence that the cremation place was in a cultivated area.

Both deceased had been affected by non-specific signs of stress (cribra cranii, cribra orbitalia, periosteal new bone formation). These signs of non-specific stress may be caused by anemia [76], rickets and osteomalacia [77, 78], scurvy [79, 80], or respiratory infections [81].

The bronze dress elements in Urn 2 suggest a female gender of the subadult; the child was cremated with dress fittings and jewelry, which suggests an average, or better than lowest position within the Late Bronze Age social stratification. Beads made out of bronze wire coils were commonly worn as part of hair ornaments, necklaces and belt fittings throughout the Bronze Age, often as part of women’s dress [82]. The health status of the individuals can be interpreted as indicators for malnutrition and disease in Bronze Age communities which have been observed in similar Late Bronze Age grave fields in the area (e.g. Wiltschke-Schrotta & Renhart [83], Renhart [84]).

### Funerary rituals

Cremation on a funerary pyre most likely took place at a different place than where the urns were buried. One pyre appears to have been built for each person, and the pyre place seems to have been re-used; this would explain the presence of more or less one person per urn, but with the occasional inclusion of body parts of a person that had been previously cremated at the same site. Urn 1 contained a second axis and two teeth that likely had been missed from the previous pyre.

Burning temperatures of over 800°C were reached and affected both the human body and the bronze artifacts, which were heavily fragmented and commingled. This suggests that the body was burnt in dressed and adorned state. Burning damage on animal bones suggest they were present on the pyre and (un)intentionally collected and placed in the urn. The slightly charred sheep or goat scapula implies to a meat-bearing bone roasted on the fire as a meat offering [85, 86]. Cremated human remains commingled with burnt animal bones is still an underrepresented observation in archaeological studies, although some studies indicated that burnt animal bones may frequently occur in urn burials in different geographical regions and over different periods from the Bronze Age to the Roman period [30, 87, 88]. We believe that the underrepresentation in archaeological studies is likely a technical bias as faunal diaphyseal fragments may often be unrecognized during the anthropological analysis, especially in strongly fragmented cremations (e.g. Wahl [89]). We highlight the importance of including the expertise of both human and animal osteologist, and refer to histological analysis in critical cases [90, 91]. Threshing residues recovered amongst the archaeobotanical remains were likely used as fire accelerant, while crops and blue elderberry seeds may be traces of food offerings. The charred plant material may also be of earlier, domestic origin unintentionally collected together with the cremated remains.

The gathering of cremated human remains for deposition into the urn aimed to recover the full body. Urn 1 and Urn 2 showed significant differences in terms of fragmentation, which is predominantly associated with the cremation process, soil pressure and excavation techniques. Parabolic heat fractures on diaphyseal fragments were clearly visible on the CT scans, indicating that fragments were already separated from each other, but were held together by the surrounding soil. To recover these fragments without destroying form information, using Paraloid is advantageous. The fragments of Urn 1 were larger on average and the degree of fragmentation was lower, which is clearly visible in the amount of identified bone fragments. The higher degree of fragmentation in Urn 2 may relate to the young age of the cremated individuals. However, the detailed sex and age-at-death estimation of the individual in Urn 1 showed the benefit of using conservation techniques during the excavation of cremated remains.

The total weight of cremated remains with 1150 g for the adult female from Urn 1 and 700 g for the child in Urn 2 is less than the expected range for modern full body cremations [38, 91, 92]. The high weight of the subadult cremation in our study, compared to other archaeological studies [e.g. 9, 84, 93] is remarkable. As bones were already visible at the top of the filling of urn 1 we cannot exclude that some remains have not been recovered from the excavation site. Fragments smaller than 2 mm represented approximately 10 to 20% of the total cremation weight in our study. Washing cremated remains in sieves with large mesh size loses the small fraction and therefore dramatically biases the recovery. These fragments are not significant for morphological analysis, but in terms of cremation weight, especially for highly fragmented subadult cremations, this strongly biases how we interpret ritual practices of selecting body parts or whole bodies for burial [94, 95]. A better proxy to identify a purposive sample of certain body areas may be the ratios of the bone weights of different body areas: The percentages would be 18.2% for the skull, 23.1% for the axial skeleton, 20.6% for the upper limbs, and 38.1% for the lower limbs [96]). All body areas are affected similarly by taphonomic processes, but these values may be lower for the axial skeleton in cremated remains and higher for the extremities as dense skeletal parts better survive the cremation process than bones with a highly spongy compound like the pelvis [96]. The ratio of collected bones in Urn 1 is similar to recovered body regions in modern cremations. Only the upper and lower limb categories contained less bone material than expected, but if the categories diaphyses, epiphyses, and autopodia are included into the limbs section, the ratios are remarkably close to modern cremations. Urn 2 showed a different picture with an overrepresentation of cranial and leg fragments and an underrepresentation of arm and torso fragments, probably due to different body proportions of subadults; all body regions were present.

Whilst gathering the cremated remains, fragments of dress, jewelry and meat offerings were also placed in the urn, again with no particular selection visible in the archaeological record. The presence of tiny droplets from bronze artifacts that would have been difficult to gather separately suggests that some pyre debris with artifact remains was scooped up and deposited in the urn.

The distribution of cremated remains within the urn suggests no particular anatomical order, but some movement of the urn after the gathering, as the urn was carried away. Contrary to other studies which observed a placement of the human remains in an anatomical order [9], there was no order observable in our case. The urns were deposited in a small pit that was not visible at the excavation and filled with soil–soil that in the case of Urn 1 contained unburnt animal and charred plant remains from the surrounding fill, whereas Urn 2 lacked plant residues entirely, except for some charcoal from the pyre.

Geochemical results are consistent with the selection of bone fragments for the urn, and the collection and dumping of remaining pyre materials into the grave pit, around the urn. Elevated Cu, relative to the other samples, was present above Urn 1 and below Urn 2. The inclusion of inorganic artifacts or costume with the body during cremation can contribute additional trace and rare-earth elements. Cu enrichment in some samples, and overall variability, is consistent with the recovery of tiny bronze droplets during micro-excavation. Furthermore, increased Al, Ca, Fe, K, Mg, Mn, and P around the urns fit with the interpretation that the presence of unburnt bones were part of unintentionally collected settlement debris.

High values for Al, Fe, K, Mg, Mn, P and Zn would be expected with the presence of ash and other burnt biomass [97–101]. Other earlier studies of unburnt bodies found enrichment of Al, Ca, K, Mg, Na, Ni, and Zn [102–104]. High values and high standard deviations for Ca and P would be expected in inhumation burials in general [105, 106]. Carbon, which would also be expected, was not determined by the testing procedures used for this study.

Concentrations for most elements are lower than expected for samples from prehistoric settlements in Central Europe [107–109]. Ca is the exception to this finding. Levels of P are likewise lower than settlement data, but similar to values from historic cemeteries in Poland [110]. Lower than typical values are partly expected due to cremation, especially when the remains are placed in an urn. Incompletely burnt bones in sediments have been found to decompose and leave chemical signatures similar to inhumations [111].

Applying a multi-method approach on prehistoric urn burials significantly increases our understanding on funerary rites in past populations. Furthermore, it facilitates disentangling taphonomic effects from intentional manipulation of the burnt bones after cremation. This helps to avoid over-interpreting the data as in the archaeological context a small cremation weight is often seen as evidence for pars-pro-toto [112]. The two urns discussed in this paper provide insights into the Bronze Age at the human scale, tracing each step of the burial ritual. The case study points out the possibilities and advantages of the applied methods and is beneficial if applied to larger samples. Consequently, we provided guidelines as S2 Text and a summary about the best practices to analyze prehistoric urn burials in Table 7.

**Table 7:** Best practices to analyze urn burials in archaeology. More details can be found in S2 Text.

Excavation on site	Recovery of the urn en-bloc
	Taking samples of the pit filling for archaeobotanical analysis [66] and soil chemistry [67]
**Radiology**	CT scans for virtual recording of the original urn burial
	MIP and automatic segmentation using thresholds for first impressions on the urn context
	Manual segmentation of diagnostic elements
**Micro-excavation**	Moisturizing of the soil prior micro-excavation
	Micro-excavation in 1–2 cm layers depending on fragmentation size
	Consolidation of diagnostic elements with Paraloid
	Washing of not-consolidated bones in water buckets using sieves with different mesh size
	Recovery of plant residue washed off the bones using sieves with a mesh size of 0.5 mm and 1 mm
	Cleaning consolidated bones with cotton swabs and acetone
	Separating human and animal bones
**Anthropological analysis**	Following guidelines for the best practice to analyze cremation burials (e.g. Brickley & McKinley [36], Jaskulska [37]
	Separating single layers into the sieve fractions larger 10 mm, 5–10 mm, 2–5 mm, smaller 2 mm
	Recording of present elements, anatomical structures, bone weight, etc.
	Sex determination using morphological and metric methods
	Age determination based on standard methods (see Buikstra & Ubelaker [113], Schaefer et al. [43]) and/or TCA
	Sorting out probable animal bones
**Zooarchaeological analysis**	Determination of animal species if possible
	Sex and age at death assessment
	Analysis of taphonomic signatures
**Archaeobotanical analysis**	Recovery of plant residue from the soil of the grave pit and the urn filling using the floatation method [66]
	Identification of plant species
**Chemical analysis**	Soil chemistry of soil samples from in and outside the urn
	Isotope analysis (e.g. Sr for mobility [52])
	C14 dating of completely calcined bone fragments and/or plant residue
**Archaeological analysis**	Analysis of stratigraphical layers of the urn and pit
	Ceramic analysis and pyre/grave good analysis
	Combining the results of the single analyses and overall interpretation of the urn burial

## Conclusion

This study showed that attention to the full contextual information of urn burials provide much more information on Bronze Age lifeways and rituals than the urns and cremated bones alone. CT scanning provided a first digital glance into the urns, but in our case, it supported rather than replaced careful micro-excavation and analysis. Archaeobotany, zooarchaeology and geochemistry allow further insights into the cultural context, the burning process, funerary rites, and deposition of urns. Micro-excavation of the urn contents with careful treatment of the cremated remains are essential, using the smallest possible sieve sizes to maximize recovery of bone fragments. Fragile diagnostic elements should be stabilized before removal from the sediment matrix. The application of this approach ensures that interpretations of prehistoric burial practices are based on the maximal amount of archaeological context data.

## Supporting information

S1 Fig Distribution of body areas in Urn 1. (PDF)

S2 Fig Distribution of body areas in Urn 2. (PDF)

S1 Appendix Sex determination and age at death estimation of the individuals from Urn 1 and Urn 2. (DOCX)

S2 Appendix Catalogue of finds. (PDF)

S1 Dataset Raw data of the anthropological results. (CSV)

S1 Text Time investment. (PDF)

S2 Text Best practices to analyze prehistoric urn burials. (PDF)
