# Supplementary material for: More than urns: A multi-method pipeline for analyzing cremation burials
Source: PLoS One. 2023 Aug 30;18(8):e0289140. doi: 10.1371/journal.pone.0289140 (PMC10468036; doi:10.1371/journal.pone.0289140)

S1: Distribution of body areas in Urn 1

Figure 1: a) horizontal distribution of body areas, b) vertical distribution of body areas (weight), c) vertical distribution of body areas (ratios) of urn 1.

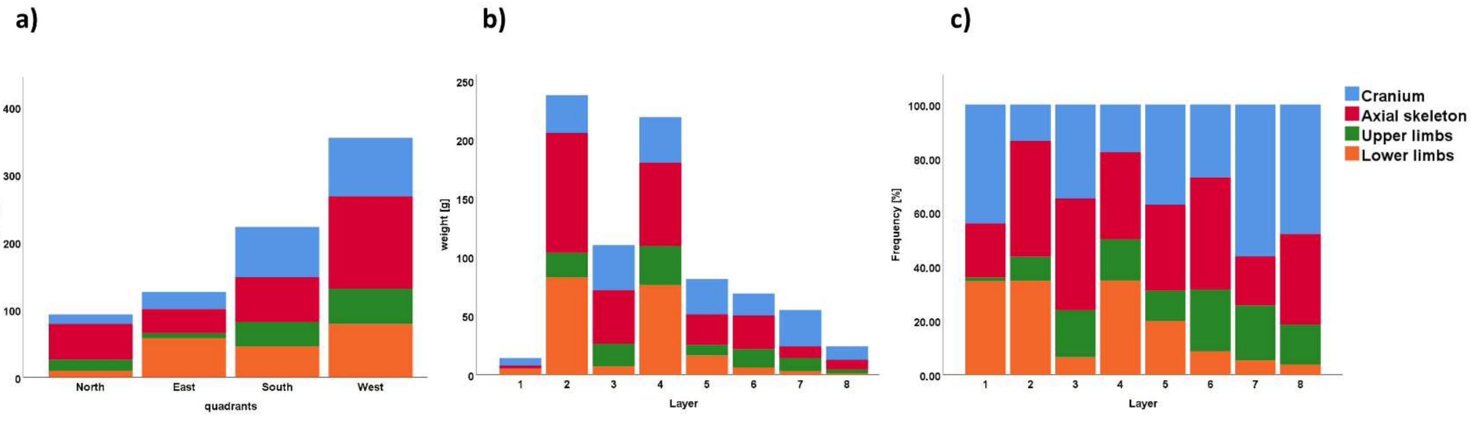

Supplement: S1 Fig — (PDF) [file pone.0289140.s001.pdf]
