# Supplementary material for: More than urns: A multi-method pipeline for analyzing cremation burials
Source: PLoS One. 2023 Aug 30;18(8):e0289140. doi: 10.1371/journal.pone.0289140 (PMC10468036; doi:10.1371/journal.pone.0289140)
Supplement: S1 Appendix — (DOCX) [file pone.0289140.s003.docx]

# **S3: Sex determination and age at death estimation of the individuals from Urn 1 and Urn 2.**

**Urn1**

**Sex determination**

**Morphologic sex determination after Ferembach & Schwidetzky [1], Buikstra & Ubelaker [2], and Walker [3]**

(0=Not observable / 1=Female / 2=Prob. Female / 3=Ambiguous / 4=Prob. Male / 5=Male)

| Cranium | | | |
| --- | --- | --- | --- |
| Feature | **Score** | **Feature** | **Score** |
| Nuchal crest | 1 | Prominence of glabella | 0 |
| Mastoid process | 1 | Mental eminence | 0 |
| Supraorbital margin | 0 | Gonion | 1 |
| Supraorbital ridges | 0 | Flexion of ramus | 0 |
|  |  |  |  |
| Pelvis | | | |
| Feature | **Score** | **Feature** | **Score** |
| Ventral arc | 0 | Sacrum | 2 |
| Subpubic concavity | 0 | Obturator foramen | 0 |
| Ischiopubic ramus | 0 | Greater sciatic notch | 2 |
| Overall shape/size | 0 | Acetabulum | 2 |

**Conclusion: probably female**

**Metric sex determination after Cavazzuti et al. [4] (adapted)**

| Element | Measurement [mm] | Estimated sex | Probability |
| --- | --- | --- | --- |
| Mandible: Condyle width | 15.8 | ambiguous | 53.8 (f) |
| Femur: vertical head diameter | 38.4 | ambiguous | 71.5 (f) |
| Radius: maximal head diameter | 15.29 | female | 99.3 (f) |

**Conclusion: female**

**Final conclusion: female**

**Age estimation**

**Epiphyeal fusion**

Union of secondary centers of ossification (after Scheuer & Black 2000). Scores: 0=Not observable / 1=Open (not fused) / 3=fused (not obliterated). In case there is a sexual dimorphism in the fusion times, the fusion times are marked with M (male) and F (female).

| **Bone** | **Epiphyses** | **Score** | **Open** | **Partially fused** | **Fused** |
| --- | --- | --- | --- | --- | --- |
| Frontal | Fusion of 2 halves of frontal bone | 3 | ≤2 yrs |  | **≥9 mths** |
|  | Obliteration of metopic suture (generally) | 3 | ≤4 yrs |  | **≥2 yrs** |
| Sphenoid | Lesser wings to sphenoid body | 0 | ≤1 mth |  | ≥5 fetal mths |
|  | Pre sphenoid to post sphenoid | 0 | ≤2 mths |  | ≥8 fetal mths |
|  | Greater wings to sphenoid body | 3 | ≤12 mths |  | **≥1 mth** |
|  | Foramen ovale (Greater wing) | 0 | ≤6 mths |  | ≥1 mth |
| Temporal | Tympanic ring to temporal squama | 0 | ≤1 mth |  | ≥9 fetal mths |
|  | Petromastoid to Squamotympanic | 3 | ≤12 mths |  | **≥9 fetal mths** |
| Occipital | Supra-occipital to interparietal squama | 3 | ≤5 fetal mths |  | **≥5 fetal mths** |
|  | Superior median suture | 3 | ≤11 mths |  | **≥5 mths** |
|  | Sutura mendosa | 0 | ≤1.5 yrs |  | ≥5 mths |
|  | Partes laterales to squama | 0 | ≤4 yrs |  | ≥1 yr |
|  | Hypoglossal canal (pars laterales) | 3 | ≤4 yrs |  | **≥1.5 yrs** |
|  | Partes laterales to pars basilaris | 0 | ≤7 yrs |  | ≥3 yrs |
| Mandible | Mandibular symphysis | 3 | ≤8 mths |  | **≥3 mths** |
| Sternum^1^ | Sternebrae 2-4 | 0 | <16 yrs |  | >11 yrs |
|  | Sternebra 1-mesosternum | 0 | <20 yrs |  | >15 yrs |
| Ribs^1^ | Head | 3 | <25 yrs |  | **>17 yrs** |
| Vertebrae^4^ | Neural arches of C3-L5 | 3 | ≤2 yrs |  | **≥6 mths** |
|  | Neural arches of C2 | 0 | ≤4 yrs |  | ≥3 yrs |
|  | Neural arches of C1 | 0 | ≤5 yrs |  | ≥4 yrs |
|  | Neural arches to centrum (C3-L5) | 3 | ≤2 yrs |  | **≥6 mths** |
|  | Dens to neural arch (C2) | 0 | ≤4 yrs |  | ≥3 yrs |
|  | Centrum to neural arch (C2) | 0 | ≤6 yrs |  | ≥4 yrs |
|  | Neural arch to anterior bar (C1) | 0 | ≤5 yrs |  | ≥4 yrs |
|  | Ossiculum terminale of dens | 3 | ≤13 yrs |  | **≥11 yrs** |
|  | Rings | 3 | M: <16.5 yrs  F: <14 yrs |  | >19 yrs  **>18 yrs** |
| Sacrum | Lateral element to neural arch | 3 | ≤5 yrs |  | **≥2 yrs** |
|  | Wing (lat. element + neural arch) to centrum | 3 | ≤6 yrs |  | **≥2 yrs** |
|  | S2-S4^2^ | 0 | <17 yrs | <23 yrs | >17 yrs |
|  | S1-S2^2^ | 0 | <17 yrs | <33 yrs | >17 yrs |
| Pelvis | Ischiopubic ramus | 3 | ≤11 yrs |  | **≥5 yrs** |
|  | Acetabulum^1^ | 3 | M: <17 yrs  F: <15 yrs |  | >14 yrs  **>11 yrs** |
|  | Iliac crest^3^ | 0 | M: <20 yrs  F: <16 yrs | 14-23 yrs | >17 yrs  >17 yrs |
|  | Ischial Tuberosity^1^ | 3 | <23 yrs |  | **>19 yrs** |
| Clavicle | Medial (sternal)^3^ | 0 | M: <25 yrs  F: <23 yrs | 17-30 yrs  16-33 yrs | >21 yrs  >20 yrs |
|  | Lateral (acromial)^1^ | 0 | M: <20 yrs  F: <20 yrs |  | >19 yrs  >19 yrs |
| Scapula^1^ | Acromion | 3 | <20 yrs |  | **>18 yrs** |
|  | Coracoid process | 0 | <17 yrs |  | >15 yrs |
|  | Medial border | 0 | <23 yrs |  | >19 yrs |
|  | Inferior angle | 0 | <23 yrs |  | >19 yrs |
|  | Glenoid epiphysis | 3 | <20 yrs |  | **>17 yrs** |
| Humerus^1^ | Greater and lesser tubercles to head | 0 | ≤6 yrs |  | ≥2 yrs |
|  | Proximal end | 0 | M: <20 yrs  F: <17 yrs |  | >16 yrs  >13 yrs |
|  | Distal end | 3 | M: <17 yrs  F: <15 yrs |  | >12 yrs  **>11 yrs** |
|  | Medial epicondyle | 0 | M: <16 yrs  F: <15 yrs |  | >14 yrs  >13 yrs |
| Radius^1^ | Proximal end | 3 | M: <17 yrs  F: <13 yrs |  | >14 yrs  **>11.5 yrs** |
|  | Distal end | 3 | M: <20 yrs  F: <17 yrs |  | >16 yrs  **>14 yrs** |
| Ulna^1^ | Proximal end (Olecranon) | 3 | M: <16 yrs  F: <14 yrs |  | >13 yrs  **>12 yrs** |
|  | Distal end | 3 | M: <20 yrs  F: <17 yrs |  | >17 yrs  **>15 yrs** |
| Hands^1^ | MC 1 | 3 | M: <16.5 yrs  F: <14.5 yrs |  | >16.5 yrs  **>14.5 yrs** |
|  | MC 2-5 | 3 | M: <16.5 yrs  F: <15 yrs |  | >16.5 yrs  **>14.5 yrs** |
|  | Proximal and middle phalanges | 3 | M: <16.5 yrs  F: <14.5 yrs |  | >16.5 yrs  **>14 yrs** |
|  | Distal phalanges | 3 | M: <16 yrs  F: <13.5 yrs |  | >16 yrs  **>13.5 yrs** |
| Femur^1^ | Head | 3 | M: <19 yrs  F: <16 yrs |  | >14 yrs  **>12 yrs** |
|  | Greater trochanter | 0 | M: <18 yrs  F: <16 yrs |  | >16 yrs  >14 yrs |
|  | Lesser trochanter | 0 | <17 yrs |  | >16 yrs |
|  | Distal end | 0 | M: <20 yrs  F: <18 yrs |  | >16 yrs  >14 yrs |
| Tibia^1^ | Proximal end | 3 | M: <19 yrs  F: <17 yrs |  | >15 yrs  **>13 yrs** |
|  | Distal end | 0 | M: <18 yrs  F: <16 yrs |  | >15 yrs  >14 yrs |
| Fibula^1^ | Proximal end | 0 | M: <20 yrs  F: <17 yrs |  | >15 yrs  >12 yrs |
|  | Distal end | 0 | M: <18 yrs  F: <15 yrs |  | >15 yrs  >12 yrs |
| Feet^1^ | Calcaneus | 0 | M: <20 yrs  F: <16 yrs |  | >18 yrs  >15 yrs |
|  | MT 1 | 0 | M: <18 yrs  F: <15 yrs |  | >16 yrs  >13 yrs |
|  | Mt 2-5 | 0 | M: <18 yrs  F: <13 yrs |  | >16 yrs  >11 yrs |
|  | Proximal phalanges | 0 | M: <16 yrs  F: <15 yrs |  | >14 yrs  >13 yrs |
|  | Distal phalanges | 0 | M: <16 yrs  F: <13 yrs |  | >14 yrs  >11 yrs |

1. Scheuer and Black (2000)

2. McKern and Stewart (1957)

3. Webb and Suchey (1985)

4. Albert and Maples (1995)

**Age: >19 years**

**Iliac auricular surface:**

Lovejoy [5]: phase V (40-44 yrs)

Osborne correction [6]: phase IV (20-75 yrs)

**Age: 20-75 years**

**Transitional analysis (cranial sutures & iliac auricular facet) after Boldsen et al. [7].**

| Cranial sutures | | |
| --- | --- | --- |
| Feature | **Score min.** | **Score max.** |
| Pterica | 1 | 1 |
| Obelica | 1 | 1 |
| Asterica | 1 | 1 |
| Interpalatine | 0 | 0 |
| zygomatico-maxillary | 0 | 0 |
|  | | |
| Iliac auricular surface | | |
| Feature | **Score min.** | **Score max.** |
| sup. topography | 3 | 3 |
| inf. topography | 2 | 2 |
| sup. characteristics | 3 | 3 |
| apical characteristics | 3 | 3 |
| inf. characteristics | 4 | 4 |
| inf. texture | 2 | 2 |
| sup. exostoses | 0 | 0 |
| inf. exostoses | 0 | 0 |
| post. exostoses | 0 | 0 |

**Age estimation: range: 23-35 years; most likely age (corr.) 23 years**

**Tooth eruption (after AlQahtani et al [8])**

Teeth were recorded using the FDI system. Only tooth roots were present. All upper permanent and both lower right PMs were recovered (14, 15, 24, 25, 34, 35). Additionally, the tooth sockets of 31, 32, 33, 41 could be evaluated, and the left lower M1 and M2 (36, 37) were present. All teeth were completely erupted.

**Age estimation: > 14.5 years**

**Tooth cementum analysis**

Two PM roots (FDI: 34,35) were used for TCA. We used an adapted version of the protocols published by Wittwer-Backofen et al [9] and Naji et al [10].The tooth layers were moderately preserved but the lines were clearly visible at several different positions (Fig. 1).

| Histo ID | Layer | FDI | Age at  tooth eruption [years] | Mean cementum thickness* [µm] | SD cementum thickness [µm] | Average double line thickness** [µm] | Calculated number of double lines | Age estimate [years] |
| --- | --- | --- | --- | --- | --- | --- | --- | --- |
| 175 | 4W | 35 | 12.5 | 43.67 | 3.73 | 2.99 | 14.6 | 27.1 |
| 176 | 4S | 34 | 11.5 | 43.46 | 5.35 | 2.99 | 14.5 | 26 |

* … Mean of 3 thickness measurements on each of 3 subsequent cross sections

** … Average of the thickness of 7 double lines of 3 cross sections of specimen no. 176 (best visibility)

**Age estimation: 26.6 years ± 5 years**


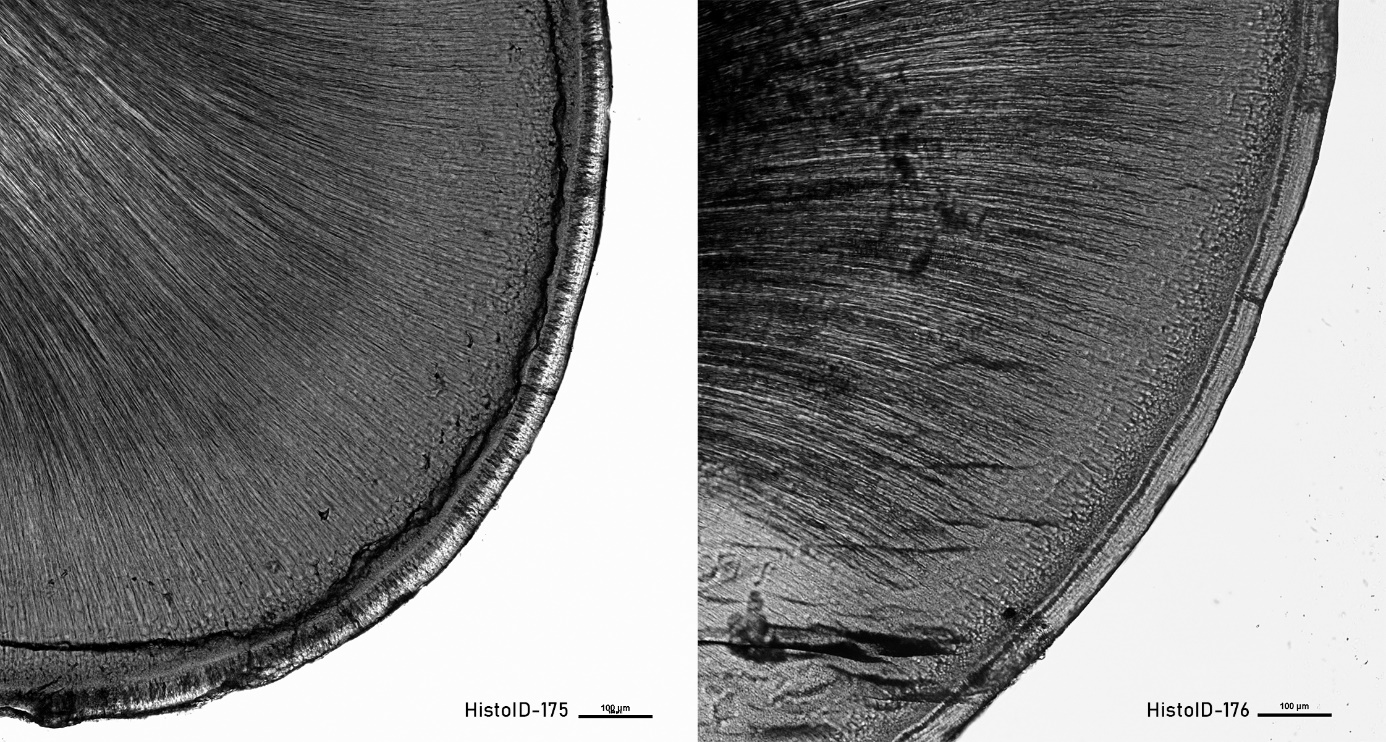
Fig. 1: Thin sections of two premolars originated from urn 1

**Final age estimation: 23-32 years (Age group: early to middle adult)**

**Urn2**

**Sex determination**

Not possible due to the young age (subadult)

**Age estimation**

**Epiphyeal fusion**

Union of secondary centers of ossification (after Scheuer & Black 2000). Scores: 0=Not observable / 1=Open (not fused) / 3=fused (not obliterated). In case there is a sexual dimorphism in the fusion times, the fusion times are marked with M (male) and F (female).

| **Bone** | **Epiphyses** | **Score** | **Open** | **Partially fused** | **Fused** |
| --- | --- | --- | --- | --- | --- |
| Frontal | Fusion of 2 halves of frontal bone | 3 | ≤2 yrs |  | **≥9 mths** |
|  | Obliteration of metopic suture (generally) | 3 | ≤4 yrs |  | **≥2 yrs** |
| Sphenoid | Lesser wings to sphenoid body | 0 | ≤1 mth |  | ≥5 fetal mths |
|  | Pre sphenoid to post sphenoid | 0 | ≤2 mths |  | ≥8 fetal mths |
|  | Greater wings to sphenoid body | 0 | ≤12 mths |  | ≥1 mth |
|  | Foramen ovale (Greater wing) | 0 | ≤6 mths |  | ≥1 mth |
| Temporal | Tympanic ring to temporal squama | 0 | ≤1 mth |  | ≥9 fetal mths |
|  | Petromastoid to Squamotympanic | 0 | ≤12 mths |  | ≥9 fetal mths |
| Occipital | Supra-occipital to interparietal squama | 0 | ≤5 fetal mths |  | ≥5 fetal mths |
|  | Superior median suture | 0 | ≤11 mths |  | ≥5 mths |
|  | Sutura mendosa | 0 | ≤1.5 yrs |  | ≥5 mths |
|  | Partes laterales to squama | 0 | ≤4 yrs |  | ≥1 yr |
|  | Hypoglossal canal (pars laterales) | 0 | ≤4 yrs |  | ≥1.5 yrs |
|  | Partes laterales to pars basilaris | 0 | ≤7 yrs |  | ≥3 yrs |
| Mandible | Mandibular symphysis | 0 | ≤8 mths |  | ≥3 mths |
| Sternum^1^ | Sternebrae 2-4 | 0 | <16 yrs |  | >11 yrs |
|  | Sternebra 1-mesosternum | 0 | <20 yrs |  | >15 yrs |
| Ribs^1^ | Head | 0 | <25 yrs |  | >17 yrs |
| Vertebrae^4^ | Neural arches of C3-L5 | 0 | ≤2 yrs |  | ≥6 mths |
|  | Neural arches of C2 | 0 | ≤4 yrs |  | ≥3 yrs |
|  | Neural arches of C1 | 0 | ≤5 yrs |  | ≥4 yrs |
|  | Neural arches to centrum (C3-L5) | 0 | ≤2 yrs |  | ≥6 mths |
|  | Dens to neural arch (C2) | 0 | ≤4 yrs |  | ≥3 yrs |
|  | Centrum to neural arch (C2) | 3 | ≤6 yrs |  | **≥4 yrs** |
|  | Neural arch to anterior bar (C1) | 0 | ≤5 yrs |  | ≥4 yrs |
|  | Ossiculum terminale of dens | 0 | ≤13 yrs |  | ≥11 yrs |
|  | Rings | 0 | M: <16.5 yrs  F: <14 yrs |  | >19 yrs  >18 yrs |
| Sacrum | Lateral element to neural arch | 0 | ≤5 yrs |  | ≥2 yrs |
|  | Wing (lat. element + neural arch) to centrum | 0 | ≤6 yrs |  | ≥2 yrs |
|  | S2-S4^2^ | 0 | <17 yrs | <23 yrs | >17 yrs |
|  | S1-S2^2^ | 0 | <17 yrs | <33 yrs | >17 yrs |
| Pelvis | Ischiopubic ramus | 0 | ≤11 yrs |  | ≥5 yrs |
|  | Acetabulum^1^ | 0 | M: <17 yrs  F: <15 yrs |  | >14 yrs  >11 yrs |
|  | Iliac crest^3^ | 0 | M: <20 yrs  F: <16 yrs | 14-23 yrs | >17 yrs  >17 yrs |
|  | Ischial Tuberosity^1^ | 0 | <23 yrs |  | >19 yrs |
| Clavicle | Medial (sternal)^3^ | 0 | M: <25 yrs  F: <23 yrs | 17-30 yrs  16-33 yrs | >21 yrs  >20 yrs |
|  | Lateral (acromial)^1^ | 0 | M: <20 yrs  F: <20 yrs |  | >19 yrs  >19 yrs |
| Scapula^1^ | Acromion | 0 | <20 yrs |  | >18 yrs |
|  | Coracoid process | 0 | <17 yrs |  | >15 yrs |
|  | Medial border | 0 | <23 yrs |  | >19 yrs |
|  | Inferior angle | 0 | <23 yrs |  | >19 yrs |
|  | Glenoid epiphysis | 0 | <20 yrs |  | >17 yrs |
| Humerus^1^ | Greater and lesser tubercles to head | 0 | ≤6 yrs |  | ≥2 yrs |
|  | Proximal end | 1 | **M: <20 yrs**  F: <17 yrs |  | >16 yrs  >13 yrs |
|  | Distal end | 1 | **M: <17 yrs**  F: <15 yrs |  | >12 yrs  >11 yrs |
|  | Medial epicondyle | 0 | M: <16 yrs  F: <15 yrs |  | >14 yrs  >13 yrs |
| Radius^1^ | Proximal end | 0 | M: <17 yrs  F: <13 yrs |  | >14 yrs  >11.5 yrs |
|  | Distal end | 0 | M: <20 yrs  F: <17 yrs |  | >16 yrs  >14 yrs |
| Ulna^1^ | Proximal end (Olecranon) | 0 | M: <16 yrs  F: <14 yrs |  | >13 yrs  >12 yrs |
|  | Distal end | 0 | M: <20 yrs  F: <17 yrs |  | >17 yrs  >15 yrs |
| Hands^1^ | MC 1 | 0 | M: <16.5 yrs  F: <14.5 yrs |  | >16.5 yrs  >14.5 yrs |
|  | MC 2-5 | 0 | M: <16.5 yrs  F: <15 yrs |  | >16.5 yrs  >14.5 yrs |
|  | Proximal and middle phalanges | 1 | **M: <16.5 yrs**  F: <14.5 yrs |  | >16.5 yrs  >14 yrs |
|  | Distal phalanges | 0 | M: <16 yrs  F: <13.5 yrs |  | >16 yrs  >13.5 yrs |
| Femur^1^ | Head | 1 | **M: <19 yrs**  F: <16 yrs |  | >14 yrs  >12 yrs |
|  | Greater trochanter | 0 | M: <18 yrs  F: <16 yrs |  | >16 yrs  >14 yrs |
|  | Lesser trochanter | 0 | <17 yrs |  | >16 yrs |
|  | Distal end | 0 | M: <20 yrs  F: <18 yrs |  | >16 yrs  >14 yrs |
| Tibia^1^ | Proximal end | 0 | M: <19 yrs  F: <17 yrs |  | >15 yrs  >13 yrs |
|  | Distal end | 1 | **M: <18 yrs**  F: <16 yrs |  | >15 yrs  >14 yrs |
| Fibula^1^ | Proximal end | 0 | M: <20 yrs  F: <17 yrs |  | >15 yrs  >12 yrs |
|  | Distal end | 0 | M: <18 yrs  F: <15 yrs |  | >15 yrs  >12 yrs |
| Feet^1^ | Calcaneus | 0 | M: <20 yrs  F: <16 yrs |  | >18 yrs  >15 yrs |
|  | MT 1 | 0 | M: <18 yrs  F: <15 yrs |  | >16 yrs  >13 yrs |
|  | Mt 2-5 | 1 | **M: <18 yrs**  F: <13 yrs |  | >16 yrs  >11 yrs |
|  | Proximal phalanges | 1 | **M: <16 yrs**  F: <15 yrs |  | >14 yrs  >13 yrs |
|  | Distal phalanges | 0 | M: <16 yrs  F: <13 yrs |  | >14 yrs  >11 yrs |

1. Scheuer and Black (2000)

2. McKern and Stewart (1957)

3. Webb and Suchey (1985)

4. Albert and Maples (1995)

**Age: 4-16 years**

**Teeth (after AlQahtani et al [8])**

Teeth were recorded using the FDI system. All upper and lower permanent incisors were completely erupted. One incisor (22) was not recovered. Additionally, the roots of a permanent first molar (not sideable) were recovered (fully erupted). The left lower PM1 (34) presented partially formed roots (approx. ¾ of the root was formed).

**Age: 8.5-14.5 years**

**Tooth cementum analysis**

One upper and one lower permanent incisor root (FDI: 11 & 41) were used for TCA.

| Histo ID | Layer | FDI | Age at  tooth eruption [years] | Mean cementum thickness* [µm] | SD cementum thickness [µm] | Average double line thickness** [µm] | Calculated number of double lines | Age estimate [years] |
| --- | --- | --- | --- | --- | --- | --- | --- | --- |
| 177 | 6W | 41 | 7.5 | 21.11 | 1.94 | 2.99 | 7.1 | 14.6 |
| 178 | 8E | 11 | 7.5 | 23.64 | 1.34 | 2.99 | 7.9 | 15.4 |

* … Mean of 3 thickness measurements on each of 3 subsequent cross sections

** … Average of the thickness of 7 double lines of 3 cross sections of specimen no. 176 (best visibility)

**Age estimation: 15.0 years ± 5 years**

**Final age estimation: 10-15 years (Age group: infans II to early adolescent)**

**References**

1. Ferembach D, Schwidetzky I, Stloukal M. Empfehlungen für die Alters- und Geschlechtsdiagnose am Skelett. Homo. 1979;30:1-32.

2. Buikstra JE, Ubelaker DH. Standards for Data Collection from Human Skeletal Remains. Davis HA, editor. Fayetteville: Arkansas Archaeological Survey; 1994.

3. Walker PL. Sexing skulls using discriminant function analysis of visually assessed traits. 2008;136(1):39-50. doi: <https://doi.org/10.1002/ajpa.20776>.

4. Cavazzuti C, Bresadola B, d'Innocenzo C, Interlando S, Sperduti A. Towards a new osteometric method for sexing ancient cremated human remains. Analysis of Late Bronze Age and Iron Age samples from Italy with gendered grave goods. PLOS ONE. 2019;14(1):e0209423.

5. Lovejoy CO, Meindl RS, Pryzbeck TR, Mensforth RP. Chronological Metamorphosis of the Auricular Surface of the Ilium: A New Method for the Determination of Adult Skeletal Age at Death. American Journal of Physical Anthropology. 1985;68:15-28. doi: 10.1002/ajpa.1330680103

6. Osborne DL, Simmons TL, Nawrocki SP. Reconsidering the Auricular Surface as an Indicator of Age at Death. Journal of Forensic Sciences. 2004;49(5):1-7.

7. Boldsen JL, Milner GR, Koningsberg LW, Wood JW. Transition analysis: a new method for estimating age from skeletons. In: Hoppa RD, Vaupel JW, editors. Paleodemography: Age Distributions from Skeletal Samples. 31. Cambridge: Cambridge University Press; 2002. p. 73-106.

8. AlQahtani SJ, Hector MP, Liversidge HM. Brief communication: The London atlas of human tooth development and eruption. American Journal of Physical Anthropology. 2010;142(3):481-90. Epub 2010/03/24. doi: 10.1002/ajpa.21258. PubMed PMID: 20310064.

9. Wittwer-Backofen U, Gampe J, Vaupel JW. Tooth cementum annulation for age estimation: results from a large known-age validation study. American Journal of Physical Anthropology. 2004;123(2):119-29. doi: 10.1002/ajpa.10303. PubMed PMID: 14730646.

10. Naji S, Colard T, Blondiaux J, Bertrand B, d'Incau E, Bocquet-Appel JP. Cementochronology, to cut or not to cut? International Journal of Paleopathology. 2016;15:113-9. Epub 20140602. doi: 10.1016/j.ijpp.2014.05.003. PubMed PMID: 29539545.
