# Supplementary material for: More than urns: A multi-method pipeline for analyzing cremation burials
Source: PLoS One. 2023 Aug 30;18(8):e0289140. doi: 10.1371/journal.pone.0289140 (PMC10468036; doi:10.1371/journal.pone.0289140)
Supplement: S2 Appendix — (PDF) [file pone.0289140.s004.pdf]

S5: Catalogue of finds

Pictures of the finds are available in Fig. 1 and Fig. 2. of supporting information 3.

Urn 1.1: large ceramic vessel used as urn, 30-60% preserved, medium fine, brown-grey ware with small stones (up to 5 mm), smoothed surface, lower part decorated with comb strokes, base c. 110 mm Ø, body c. 340 mm Ø, wall thickness 6-7 mm

Urn 1.2: sherd of a large ceramic vessel found under the urn, fine to medium, orange-brown to grey micaceous ware with small stones (up to 2 mm), well smoothed, decorated with wide cannelure, body c. 300 mm Ø, wall thickness 4-7 mm

Urn 1.3: bronze ring, fire affected, complete, 35 × 31 × 3.5 mm, 7.4 g, oval cross section, casting thorne from bivalve casting mold with 1.5 mm misalignment, found within the south western part of the urn in layer 5

Urn 1.4: twisted bronze wire ring (Noppenring), fire affected, 2.5 loops preserved, c. 27 × 19 × 4 mm, oval wire 0.9-1.3 mm Ø, found within the south western part of the urn in layer 5

Urn 1.5: 8 fragments of a bronze wire, fire affected, c. 40 mm, c. 2 mm round Ø, c. 0.2 g, found outside the urn towards the north

Urn 1.6: three fragments of a bronze wire, fire affected, c. 17 × 6 × 2.3 mm, c. 2 mm round Ø, found outside the urn towards the east

Urn 1.7: 3 fragments of a bronze wire, c. 11 × 3.2 mm, c. 3.2 mm round Ø, 0.1 g, found within the north part of the urn in layer 7

Urn 1.8: 18 small bronze droplets from artefacts molten on the pyre, c. 3 × 3 × 3 mm, c. 0.4 g, found outside the urn towards the south

Urn 1.9: bronze drop from an artefact molten on the pyre, c. 2.5 × 2.5 × 2.5 mm, <0.1 g, found outside the urn towards the north

Urn 1.10: sheet bronze fragment, fire affected, c. 7 × 6 × 0.25 mm, <0.1 g, found towards the north-east at the outside of the urn

Urn 1.11: bronze drop from an artefact molten on the pyre, c. 2.4 × 2.4 × 2.4 mm, <0.1 g, found outside the urn towards the west

Urn 1.12: bronze drop from an artefact molten on the pyre, c. 3.3 × 2.7 × 2.7 mm, <0.1 g, found in the center of the urn in layer 1

Urn 1.13: bronze drop from an artefact molten on the pyre, c. 3 × 2.5 × 2 mm, <0.1 g, found within the western part of the urn in layer 4

Urn 1.14: fragments of bronze patina, c. 3 × 2.5 × 2.5 mm, <0.1 g, found within the urn, layer 1, western part

Urn 2.1: bi-conical ceramic vessel used as urn, almost complete, dark grey medium-fine ware with small stones (up to 2 mm), polished surface covered with graphite, base 90 mm Ø, body 215 mm Ø, rim 165 mm Ø, wall thickness 6-7 mm

Urn 2.2: 30 small fragments of the wall and base of a ceramic vessel (cup?), <10 % preserved, grey-orange fine ware with small stones (up to 2 mm), well-polished, fire affected, found north of the urn

Urn 2.3: rim and wall fragments of at least one ceramic vessel (cup or bowl?), <10 % preserved, grey fine ware with few small stones (< 2 mm), polished inside covered with graphite, fire affected, 4-6 mm wall thickness, found outside and inside of the urn

Urn 2.4: four very small pottery fragments, grey, fine ware, 2-3 mm wall thickness, found in the upper 7 cm within the urn

Urn 2.5: two ceramic base fragments, orange-grey fine ware with small stones (< 1.5 mm), roughly polished, fire affected, 5-6 mm wall thickness, found within the urn 130-140 mm below the top

Urn 2.6: fragment of a bronze wire ring (armring?), fire affected, 35 × 8 mm, twisted wire 2.4 mm Ø, 1.1. g, found at the base 170-175 mm below the top of the urn

Urn 2.7: conical object made of folded sheet bronze (c. 0.4-0.5 mm thick), c. 21 × 9 × 5 mm, 0.98 g, found within the southwestern part of the urn 145 mm below the top

Urn 2.8: 14 fragments of coil beads (Spiralröllchen) made of flat to semi-circular bronze wire, fire affected, 13 loops preserved, c. 20 × 25 × 0.5-2 mm, 0.2 g, found within the southwestern part of the urn 130 mm below the top

Urn 2.9: fragments of coil beads (Spiralröllchen) made of flat bronze wire, fire affected, 4 loops preserved, c. 41 × 25 × 0.5 mm, <0.1 g, found within the west-central part of the urn 155 mm below the top

Urn 2.10: fragments of coil beads (Spiralröllchen) made of flat bronze wire, fire affected, 1.3 loops preserved, c. 4 × 3.5 × 0.5 mm, <0.1 g, found within the north-central part of the urn 165 mm below the top

Urn 2.11: bronze drop from an artefact molten on the pyre, 17 × 8 × 5.5 mm, 2.8 g, found within the western base part of the urn 170-175 mm below the top

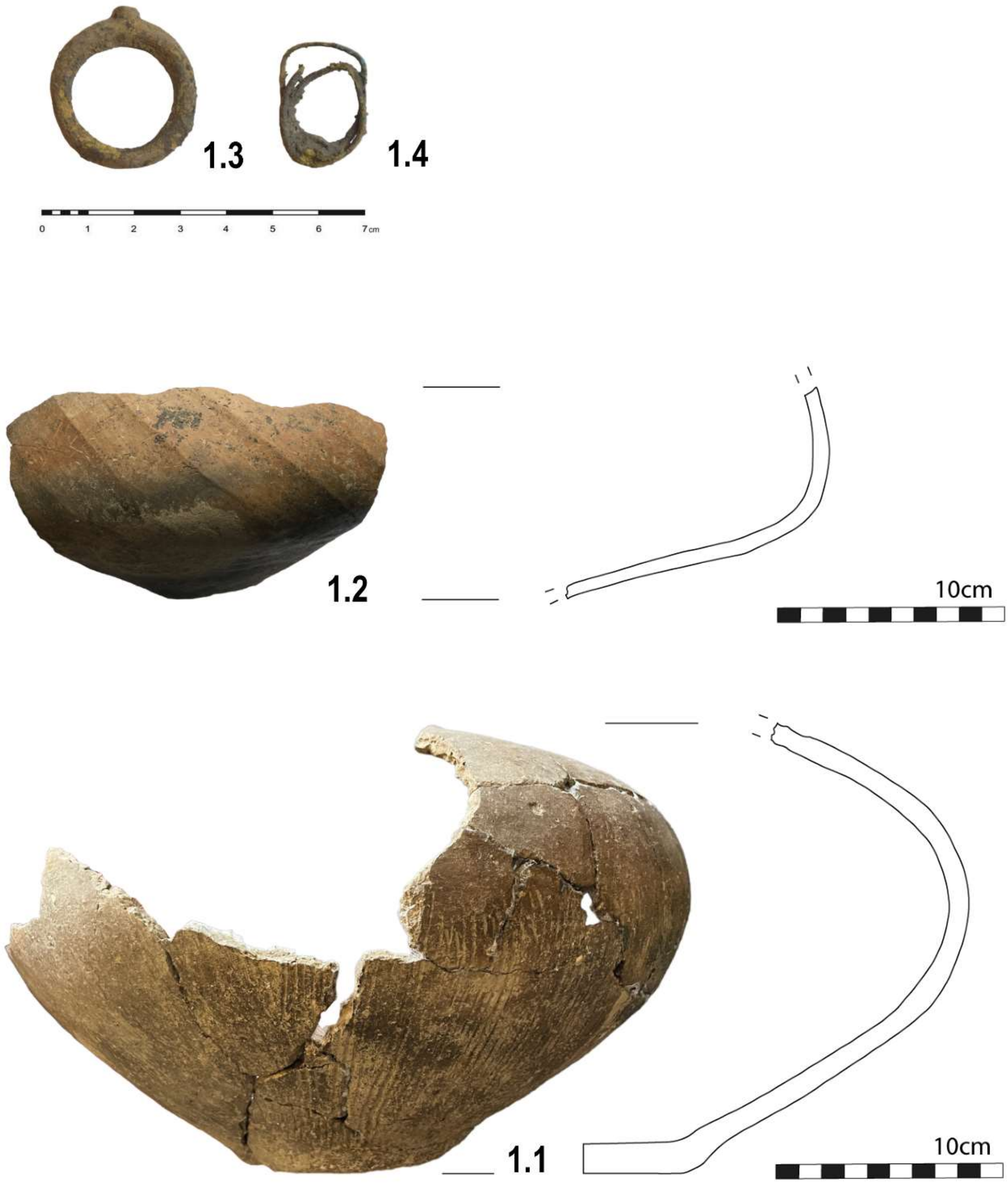

Figure 1: outline and photo of the recovered ceramic vessels from block 1 (1.1: urn 1, 1.2: sherd of a large ceramic vessel deposited under urn 1) and photo of the pyre goods (1.3: bronze ring, 1.4: twisted bronze wire ring (Noppenring)) recovered from urn 1.

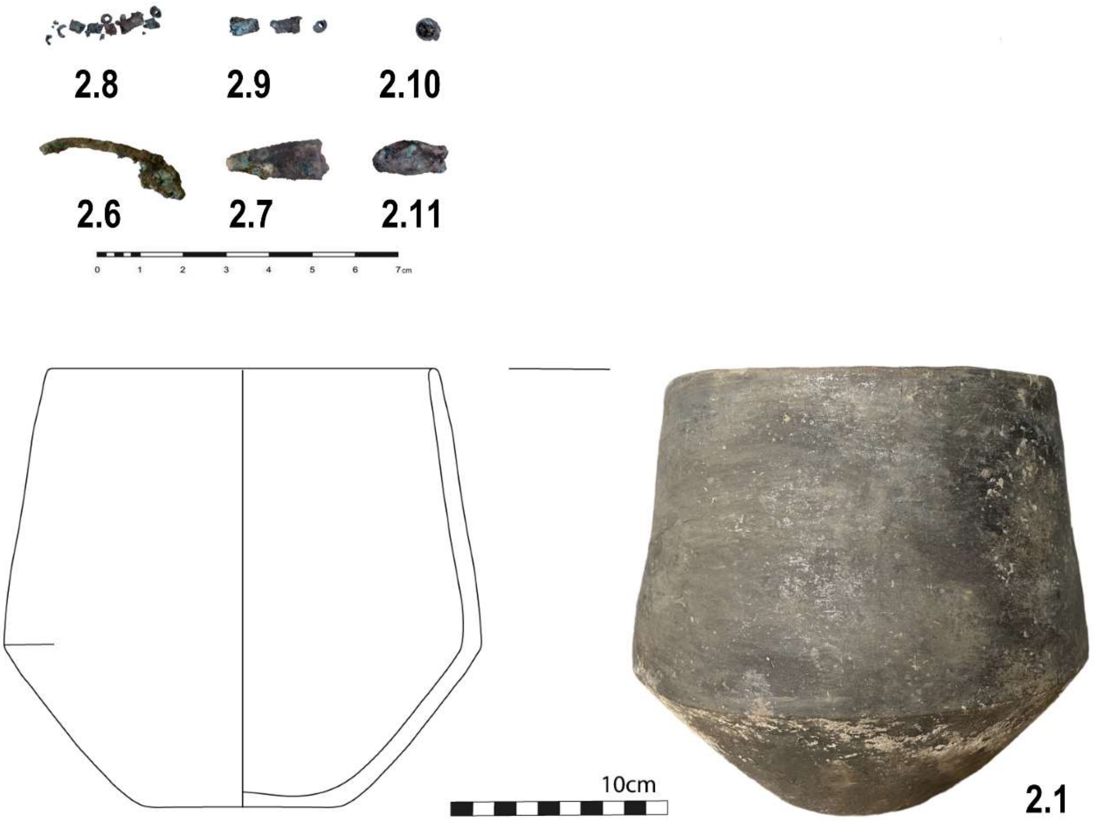

Figure 2: outline and photo of the recovered ceramic vessel urn 2 (2.1) and photo of the pyre goods (2.6: fragment of a bronze wire ring (armring?), 2.7: conical object made of folded sheet bronze, 2.8, 2.9, 2.10: fragments of coil beads (Spiralröllchen), 2.11: bronze drop from an artefact molten on the pyre) recovered from urn 2.
