# Supplementary material for: More than urns: A multi-method pipeline for analyzing cremation burials
Source: PLoS One. 2023 Aug 30;18(8):e0289140. doi: 10.1371/journal.pone.0289140 (PMC10468036; doi:10.1371/journal.pone.0289140)
Supplement: S1 Text — (PDF) [file pone.0289140.s006.pdf]

S6: Time investment

As expected, the segmentation of the CT scans and the micro-excavation of urns are time-intensive processes (Tab. 1). A first virtual analysis of the urn context can be performed within one to two hours, which makes it a quick method in comparison to a micro-excavation of the urn. A semi-manual segmentation that provides attractive data representations for public outreach, however, totaled more than 25 hours of work. The large time difference between both urns suggests that variable factors, for instance urn size, fragmentation size, and consolidation highly affect the duration of the micro-excavation. Urn 2 was only filled to a third with bone fragments, which allowed to quickly remove the soil above the cremation layer. The micro-excavation of Urn 1 was slowed down by the discovery of a charcoal-rich layer which we decided to excavate stratigraphically. Furthermore, Urn 1 contained large bone fragments which passed through several excavated layers and made the excavation more complex in comparison to Urn 2. Consolidating the bones was straight-forward as they were simply covered with the consolidant and left to dry for a few minutes. The difference in cleaning time is affected by the number of excavated layers (which were kept separated for the anthropological examination), the number of bones to clean (Urn 1 contained nearly double the amount of Urn 2), and the number of consolidated bones. Stabilized bones had to be manually cleaned with acetone and cotton swabs from the surrounding soil, because Paraloid is not water-soluble. The cleaning time could be reduced if the exposed bone surface was carefully cleaned from soil using water and cotton swabs before consolidation and recovery. The time needed for the anthropological analysis was clearly reduced if the bones were consolidated. Large bone fragments can be identified more easily and less time is needed for sorting and identifying anatomical elements. Time investment for archaeobotanical and zooarchaeological analysis was dependent on the absence and presence of material. Sorting plant residues and identifying them under the microscope is time-consuming, but revealed nearly 19,000 plant residues. In addition, bucket floatation recovered over 30 g of bone fragments smaller than 2 mm that had remained in the soil after excavation and added to the completeness of the cremated remains.

Tab. 7. workhours for selected single tasks during this study (time in [hh:mm])

**Tasks**	**Urn 1**	**Urn 2**
CT scanning	0:30	0:30
Post processing & segmentation	26:30	28:15
Micro-excavation (10 mm layer including consolidation)	119:00	68:30
Cleaning of bones and the urn	24:00	8:15
Reconstruction of the urn	9:00	1:30
Anthropological examination	10:00	16:00
Archaeobotanical analysis	20:00	0:30
Zooarchaeological analysis	2:00	-
Total time	211:00	123:30
