# Supplementary material for: More than urns: A multi-method pipeline for analyzing cremation burials
Source: PLoS One. 2023 Aug 30;18(8):e0289140. doi: 10.1371/journal.pone.0289140 (PMC10468036; doi:10.1371/journal.pone.0289140)
Supplement: S2 Text — (PDF) [file pone.0289140.s007.pdf]

Supporting information 7: Best practices to analyze prehistoric urn burials

More than in most areas of archaeology, the analysis of urn burials needs interdisciplinary collaboration. The workflow to analyze prehistoric urn burials is visible in Fig. 1. At best, an osteologist participates in the excavations to make sure that all bones are recovered. This is especially important for highly fragmented urns, as post-depositional processes such as bioturbation, rain and water table changes may dislocate tiny bone fragments. In our case, the urns were recovered during a rescue excavation. Rescue excavations are usually performed under time pressure with a limited amount of financial resources, which increases the risk of losing important details on the burials. We suggest to always recover the urn en-bloc if the burial is overall undisturbed and the urn is only slightly damaged. If CT scans of the urns are planned prior to micro-excavation, we suggest keeping the block as small as possible to reduce cone beam hardening artifacts in the scans. For a first impression of the urn context, a maximum intensity projection, and an automatic segmentation based on thresholds may be sufficient. However, to obtain 3D models of single bones, the best results can be obtained by manual segmentation. Furthermore, the recovery of the entire pit fill or a significant sample for micro-archaeological analysis, in particular archaeobotanical analysis, is indicated. Our analysis showed that charcoal-rich layers contained the largest amount of plant residues.

Figure 1: Workflow to analyze urn burials.

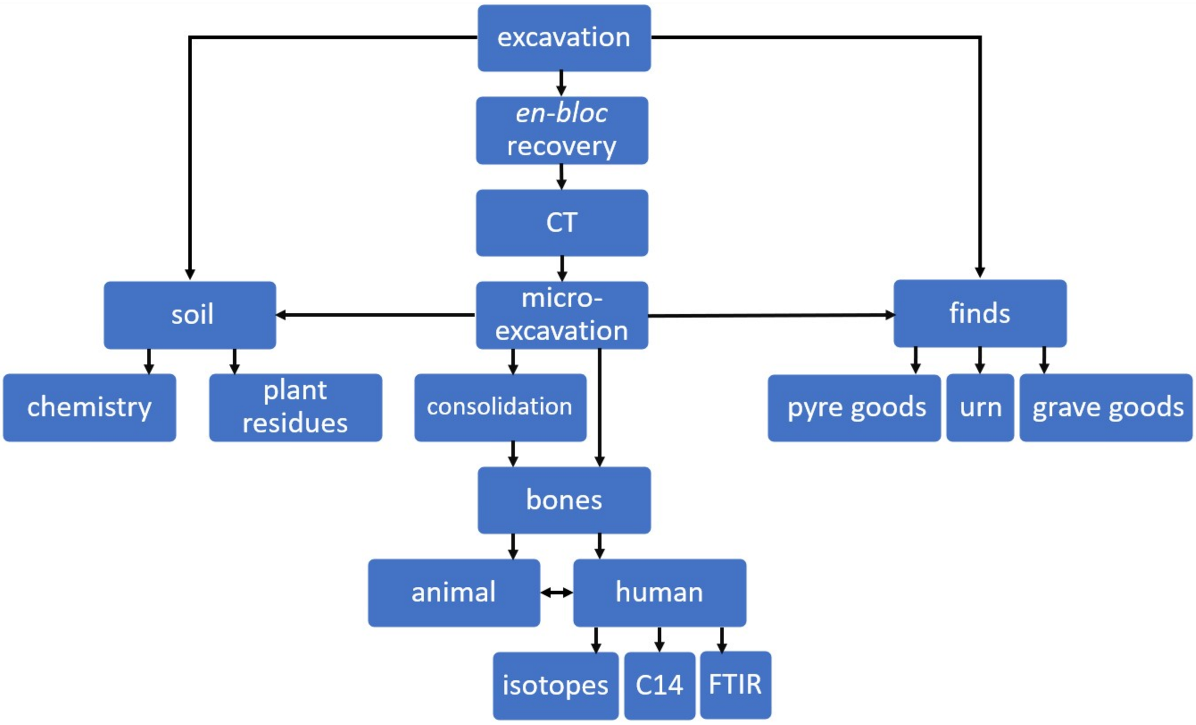

Urns recovered en-bloc can be stored for many years if there are no funds available to analyze them in detail. The soil may dry and form cracks, but, in our experience, this only has a minor impact on the cremated remains if the soil is moisturized before and during the micro-excavation. The thickness of the arbitrary excavation layers may vary based on the size of the bone fragments. If the layers are too wide, a possible order of recovered body areas may be missed. If the layers are too small, many elements fall into several layers, which causes a more difficult and time-intensive excavation. If CT scanning was not performed before excavation, we suggest starting with 1 cm layers until the layer containing the cremation is reached, and then adapting the thickness of the layers based on the bone condition. Usually, wooden tools are preferable for the micro excavation, but this is dependent on the soil. In our case, the soil contained a high amount of clay, which made an excavation with wooden tools difficult. We received the best results using dental tool kits. Especially dental probes allowed an easy removal of soil attached to bone fragments. The micro-excavation will be best performed by a trained anthropologist, who will also perform the osteological analysis. This allows in-situ measurements of diagnostic elements and the application of Paraloid on important elements. Paraloid should only be sparingly applied on elements that are of crucial importance for the osteological assessment, such as elements for a sex and age at death estimation, pathologies, or enthesial changes. Paraloid can be usually purchased as crystals which are dissolvable in acetone. We used an 8%-solution, which can be applied onto the bones with a pipette for the consolidation, and a 60%-solution for gluing bone fragments together. Before the application of Paraloid, it is important to carefully clean the bone surface with water and cotton swabs to allow a better consolidation of the bone fragments and an easier cleaning of the bones after recovery. Later on, the elements can be further cleaned using Acetone and cotton swabs. It is important to clean only small areas at once and to spare time for the acetone to evaporate again. If the bones became soaked with acetone, the Paraloid may dissolve again and the elements may fall apart. The consolidation of the bones and the cleaning process will be best performed under a fume hood to avoid breathing in the acetone fumes. Large bone fragments which have not been consolidated before recovery should be packed separately and labelled with the identified element as breakage may make it impossible to recognize the element based on the form of the fragments. We recommend leaving bones untreated for

further chemical analysis. Bones which have not been treated with Paraloid can be washed with water. We washed the bones using a bucket of water and sieves with different mesh sizes (1 cm to 1 mm).

The exact process how to wash cremated bones best is dependent on burning conditions, the fragment size, their fragility, and soil conditions. Completely calcined diaphyseal fragments can best be washed using water and a tooth brush as they are solid. Fragile fragments are best washed in sieves with a small mesh size by carefully moving the sieve into water. As plant residuals are floating, they can be recovered with a sieve with a mesh size of 0.5 mm and 1 mm. Remaining soil of the pit and urn filling shall be recovered by an archaeobotanist using the floatation method after taking samples for soil chemistry (approx. 20g per sample).

The anthropological examination follows published guidelines (e.g. Brickley & McKinley [1], Jaskulska [2]). Grosskopf [3] also provides additional advice and observations on the analysis of cremated remains. Due to inter-population differences, we suggest to use seriations of diagnostic elements for sex determination, which commonly occur in various urn burials excavated in the same area. Age at death estimation in adults is often limited to cranial sutures if the iliac auricular facet or the pubic symphysis was not preserved. If tooth roots are present in the cremation burial, we suggest performing tooth cementum analysis to narrow down the age ranges. CT scans of complete bones provide further support to assign bone fragments to single elements as the diaphyses of bones change their shape of cross sections with varying position. As animal bones are commonly commingled with the human remains, we suggest that the human osteologist collaborates with an animal bones specialist and trains aspects in zooarchaeology before the analysis. In our case, bone fragments of unusual shape and size for humans were sorted out and handed over to the zooarchaeologist for further analysis. In some cases, the zooarchaeologist could not assign fragments to animal species and handed these fragments back; asecond evaluation sometimes identified these fragments as human fragments strongly deformed by heat. Isotope analyses and C14 dating will be best performed on completely calcined elements of one fragment. This will reduce the risk of choosing two elements of two individuals by random which have not been recognized during the osteological analysis (see Sabaux et al. [4])

References

1. Brickley M, McKinley I. Guidelines to the Standards for Recording Human Remains. Southampton, Reading: BABAO; 2004.
2. Jaskulska E. Analysis of human cremains. Warsaw: University of Warsaw; 2020.
3. Grosskopf B. Cremations, more than just a little pile of ash. Anthropologischer Anzeiger; Bericht uber die biologisch-anthropologische Literatur. 2020. Epub 2020/07/25. doi: 10.1127/anthranz/2020/1249. PubMed PMID: 32706015.
4. Sabaux C, Veselka B, Capuzzo G, Snoeck C, Sengeløv A, Hlad M, et al. Multi-proxy analyses reveal regional cremation practices and social status at the Late Bronze Age site of Herstal, Belgium. Journal of Archaeological Science. 2021;132:105437. doi: https://doi.org/10.1016/j.jas.2021.105437.
